# A small RNA-guided PRC2 complex eliminates DNA as an extreme form of transposon silencing

**DOI:** 10.1016/j.celrep.2022.111263

**Published:** 2022-08-23

**Authors:** Chundi Wang, Therese Solberg, Xyrus X. Maurer-Alcalá‬, Estienne C. Swart, Feng Gao, Mariusz Nowacki

**Affiliations:** 1 Institute of Cell Biology, University of Bern, Baltzerstrasse 4, 3012 Bern, Switzerland; 2 Institute of Evolution & Marine Biodiversity, Ocean University of China, Qingdao 266003, China; 3 Laboratory of Marine Protozoan Biodiversity & Evolution, Marine College, Shandong University, Weihai 264209, China; 4 Division of Invertebrate Zoology and Sackler Institute for Comparative Genomics, American Museum of Natural History, New York, NY 10024, USA; 5 Max Planck Institute for Biology, Max Planck Ring 5, 72076 Tuebingen, Germany; 6 Key Laboratory of Mariculture (OUC), Ministry of Education, Qingdao 266003, China; 7 Laboratory for Marine Biology and Biotechnology, Qingdao National Laboratory for Marine Science and Technology, Qingdao 266237, China

**Keywords:** PRC, transposable elements, transposons, DNA elimination, piRNA, ciliates, H3K9me3, H3K27me3, heterochromatin, Piwi

## Abstract

In animal germlines, transposons are silenced at the transcriptional or post-transcriptional level to prevent deleterious expression. Ciliates employ a more direct approach by physically eliminating transposons from their soma, utilizing piRNAs to recognize transposons and imprecisely excise them. Ancient, mutated transposons often do not require piRNAs and are precisely eliminated. Here, we characterize the Polycomb Repressive Complex 2 (PRC2) in *Paramecium* and demonstrate its involvement in the removal of transposons and transposon-derived DNA. Our results reveal a striking difference between the elimination of new and ancient transposons at the chromatin level and show that the complex may be guided by Piwi-bound small RNAs (sRNAs). We propose that imprecise elimination in ciliates originates from an ancient transposon silencing mechanism, much like in plants and metazoans, through sRNAs, repressive methylation marks, and heterochromatin formation. However, it is taken a step further by eliminating DNA as an extreme form of transposon silencing.

## Highlights

• PRC2 couples Piwi-bound small RNAs to transposon elimination in the new soma
• Elimination of new and ancient transposon sequences depends on PRC2
• Loss of PRC2 leads to retention and de-repression of transposons
• Only new transposons require heterochromatin and small RNAs for their removal

## Graphical abstract

**Figure:**
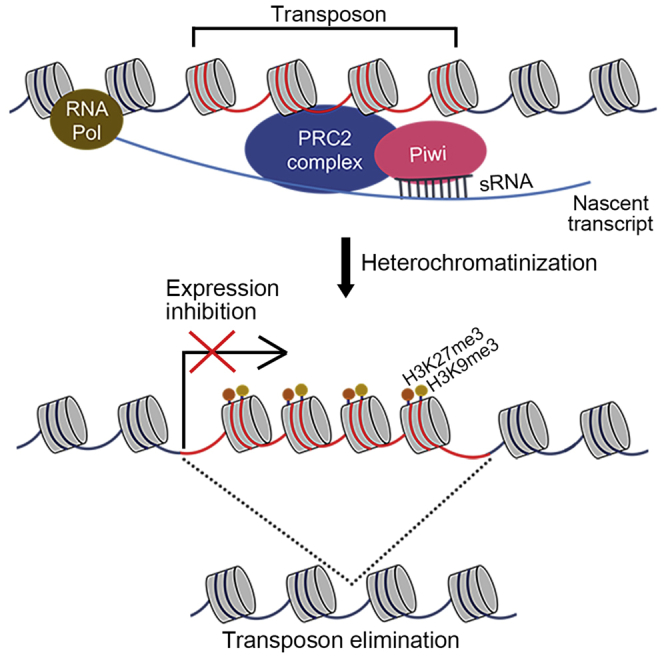

## Teaser

In animal germlines, the piRNA pathway silences transposons at the transcriptional level by deposition of repressive methylation marks. Wang et al. identify a PRC2 complex as the link between Piwi-bound small RNAs and DNA elimination as an extreme form of transposon silencing.

## Introduction

Transposable elements (TEs) are repetitive DNA sequences that have the potential to move and replicate in the genome, posing a potential threat to genome integrity when left unchecked. They are widely distributed and account for a large proportion of the genome. For instance, they may account for more than two-thirds of the human genome, two orders of magnitude greater than the number of protein-coding genes (de Koning et al., 2011). Despite that they are major drivers of evolution through their ability to modify the genomic architecture, increase genetic diversity, and regulate gene expression, they must be subject to tight control to prevent TE-induced damage to the genome (Friedli and Trono, 2015; Slotkin and Martienssen, 2007). Therefore, a diverse set of mechanisms evolved to suppress these elements. This is mostly achieved by the deposition of repressive chromatin marks during development, the best characterized of which are DNA methylation and histone H3 lysine methylation (Walter et al., 2016; Pezic et al., 2014; Sienski et al., 2012; Aravin et al., 2008; Kuramochi-Miyagawa et al., 2008).

In animal germlines, the piRNA pathway represses TEs through both transcriptional and post-transcriptional silencing mechanisms (reviewed in Ozata et al., 2019). Transcriptional repression is ensured by the histone methyltransferase SETDB1, guided by small RNA (sRNA)-Piwi complexes to target TEs in a sequence-specific manner (Sienski et al., 2012; Schultz et al., 2002). This enzyme catalyzes the trimethylation of lysine 9 on histone H3 (H3K9me3), leading to heterochromatin formation and silencing. Together with the trimethylation of lysine 27 on histone H3 (H3K27me3), they form the two best-studied heterochromatin marks. The second methylation mark is set by members of the Polycomb group (PcG) proteins, parts of large multiprotein complexes regulating developmental gene expression. One of the most well-studied PcG complexes is Polycomb Repressive Complex 2 (PRC2), which deposits H3K27me3 on target genes and is essential for development (Cao et al., 2002; Czermin et al., 2002; Kuzmichev et al., 2002; Müller et al., 2002). Although both H3K9me3 and H3K27me3 are heterochromatin marks, they are generally considered to perform vastly different functions: H3K9me3 ensures the repression of TEs, and H3K27me3 is involved in developmental gene repression.

In ciliates, a large group of single-celled eukaryotes that contain both the germline micronucleus (MIC) and the somatic macronucleus (MAC) in the same cytoplasm, elimination of transposon and transposon-derived DNA is ensured through a piRNA-like pathway, in which Piwi-bound sRNAs function as mediators in a comparison event between the germline and somatic nucleus. The transcriptionally inactive germline contains TEs, minisatellites, and tens of thousands of transposon remnants known as internally eliminated sequences (IESs), all of which must be removed during the formation of a new soma (Arnaiz et al., 2012). DNA elimination in *Paramecium tetraurelia* features both imprecise elimination of repetitive sequences (TEs and minisatellites) and precise elimination of around 45,000 IESs, derived from TEs (Arnaiz et al., 2012; Le Mouël et al., 2003). IES size correlates with their evolutionary origin and dependence on sRNAs for their excision. Recently acquired elements tend to be much longer because they are derived from evolutionarily recent TE insertions, and their recognition and elimination more strongly depend on targeting by sRNAs. Very short IESs are evolutionarily more ancient, and the cells have acquired the ability to recognize and excise them without the help of sRNAs.

During development, a class of Piwi-bound sRNAs (scnRNAs) is produced from the entire MIC genome by bidirectional transcription and cleavage of the transcript by the dicer-like enzymes Dcl2/3 (Sandoval et al., 2014; Lepere et al., 2009). After binding their Piwi partners, Ptiwi01/09, scnRNA-Ptiwi complexes enter the maternal MAC for a process known as scanning, in which they are compared with the genome of the previous generation (Mochizuki et al., 2002). The scnRNAs that find complementary sequences are removed, whereas those that are not are further transported to the new MACs, where they target cDNA sequences for elimination by a domesticated piggyBac-like transposase called PiggyMac (Pgm) (Baudry et al., 2009). The involvement of repressive chromatin marks in this process has been known for a while; however, the reports have been vague or indirect at best, and the connection between them has not been investigated. Early reports have included developmental expression of H3K9me3 and H3K27me3, which depend on scnRNAs, and an involvement of Ezl1 (a EZH2 homolog) and PtCAF1 (a RbAp46/48 homolog) in DNA elimination (Arambasic et al., 2014; Ignarski et al., 2014; Lhuillier-Akakpo et al., 2014). Recently, the *Paramecium* Ezl1 protein has been shown to possess dual-methyltransferase activity *in vitro* and methylates both H3K9me3 and H3K27me3 during development, both of which co-occur on TEs (Frapporti et al., 2019). It was further shown that depletion of Ezl1 leads to de-repression of TEs. Although Ezl1 is present in two distinct nuclei at two different stages of development, no studies have investigated its role exclusively in the new MACs. Furthermore, possible interaction partners of Ezl1 have not been reported. We therefore sought to characterize the Ezl1-containing protein complex, as well as elucidate its specific functions in both early and late development.

Here, we identify the complex of Ezl1 in the ciliate *Paramecium tetraurelia* as a prototypical PRC2 complex composed of the conserved core subunits: Ezl1 (EZH1/2), Suz12 (SUZ12), Eed (EED1), and PtCAF1 (RbAp46/48). The complex also contains two Ring-finger domain proteins, Rnf1 and Rnf2. We show that the PRC2 complex is essential for sexual reproduction and DNA elimination events, as well as the scanning process in early stages of development. Silencing of PRC2 subunits leads to downregulated expression of hundreds of genes during development, which is contrary to the typical repressive function of the complex in other eukaryotes. By modifying the timing of expression, we distinguish two independent functions of the complex: one in the maternal MAC and one in the new MACs. We show that the former is dispensable for most DNA elimination events, including imprecise elimination of TEs. Chromatin immunoprecipitation sequencing (ChIP-seq) and nucleosome profiling in the absence of PRC2 reveal a striking difference between precise and imprecise DNA elimination events at the chromatin level. We propose a model in which the precise elimination of IESs and the imprecise elimination of TEs follow two distinct pathways, the latter of which is analogous to the piRNA pathway in higher eukaryotes.

## Results

### Interaction between *Paramecium* Ezl1 methyltransferase and PtCAF1 chromatin assembly factor

**Figure 1:**
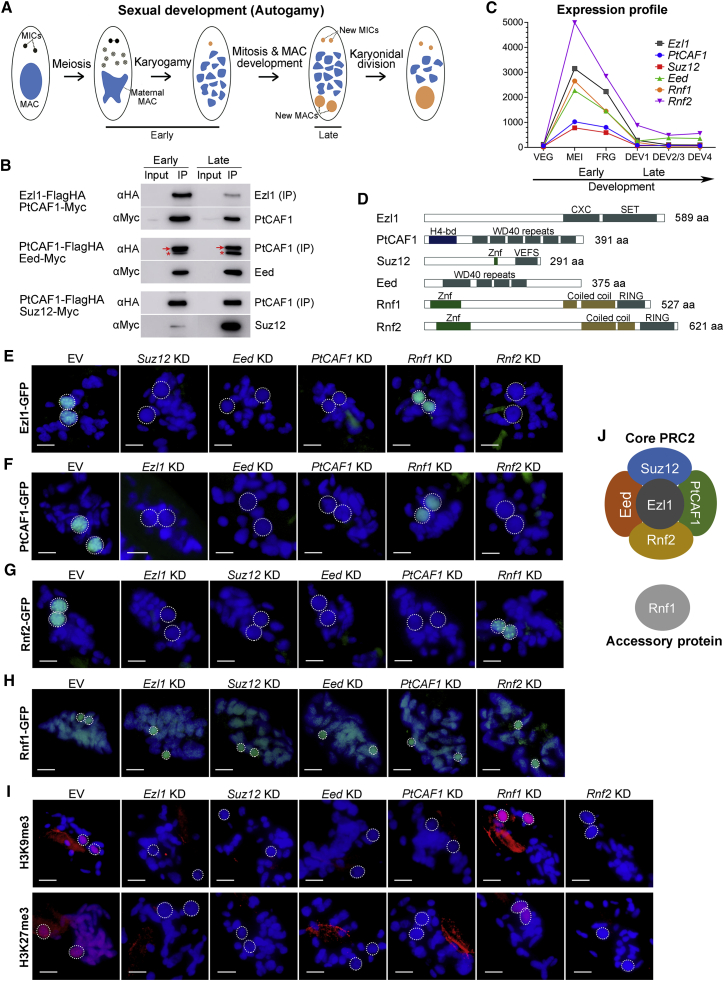
Identification of the core components of PRC2 (A) Sexual development of *Paramecium tetraurelia*. The MICs undergo meiosis to produce eight gametic nuclei, one of which performs mitosis and the haploid nuclei fuse. After two mitoses, two nuclei develop into new MICs and the other two develop into new MACs. The development of new MACs includes DNA elimination, chromosome breakage, telomere addition, and DNA amplification. The fragments of the maternal MAC gradually degrade during this process. Karyonidal division completes autogamy, with each daughter cell harboring two MICs and one MAC. (B) Western blots of HA-immunoprecipitated proteins using anti-HA or anti-Myc antibodies to detect Ezl1, PtCAF1, Eed, and Suz12. Red arrow is PtCAF1; asterisk is Eed. (C) Expression profiles based on RNA-seq data from Arnaiz et al. (2020). (D) Predicted domains and size of Ezl1, PtCAF1, Suz12, Eed, Rnf1, and Rnf2. (E) Localization of Ezl1-GFP on EV, *Suz12*, *Eed*, *PtCAF1*, *Rnf1*, and *Rnf2* KD. (F) Localization of PtCAF1-GFP on EV, *Ezl1*, *Eed*, *PtCAF1*, *Rnf1*, and *Rnf2* KD. (G) Localization of Rnf2-GFP on EV, *Ezl1*, *Suz12*, *Eed*, *PtCAF1*, and *Rnf1* KD. (H) Localization of Rnf1-GFP on EV, *Ezl1*, *Suz12*, *Eed*, *PtCAF1*, and *Rnf2* KD. (I) Immunofluorescence of H3K9me3 and H3K27me3 on EV, *Ezl1*, *Suz12*, *Eed*, *PtCAF1*, *Rnf1*, and *Rnf2* KD. (J) Components of the core PRC2 complex and its accessory protein. Green: GFP signal; dark blue: DAPI; red: immunofluorescence of H3K9me3 or H3K27me3 as specified (E–I). New MACs are indicated by white dashed circles. Scale bars: 10 μm. EV, empty vector silencing. See also Figures S1–S4 and Table S1.

Previous work from our and other groups identified PtCAF1, a homolog of RbAp46/48, and Ezl1, a methyltransferase responsible for H3K9me3 and H3K27me3, as genes involved in *Paramecium* sexual development (Ignarski et al., 2014; Lhuillier-Akakpo et al., 2014). They have been shown to be essential for the elimination of IESs and TEs, and both affect the levels of developmental H3K9me3 and H3K27me3 on depletion. Despite having nearly identical phenotypes when depleted, the connection between them has remained elusive. One explanation of their high IES retention correlation is an indirect effect, where the transcription of one is dependent on the other. If this were the case, we would see them phenocopy each other when depleted. To establish if this was the case, we expressed a PtCAF1-GFP construct in Ezl1-knockdown (KD) cells and in wild-type (WT) cells (control). In the control, PtCAF1 localized as expected: first in the maternal MAC in early developmental stages, and then in the new MACs as they appeared (Figures 1A and 1F). However, the GFP signal was not detected in Ezl1-KD cells (Figure 1F). To determine whether this was on the level of transcription or protein stability, we extracted total RNA from control and Ezl1-KD cells. A northern blot using a probe against PtCAF1 excluded an indirect effect on transcription, because the mRNA levels were unchanged in Ezl1-KD (Figure S1). The opposite was also tested (i.e., the localization of Ezl1-GFP fusion in PtCAF1-KD cells), which yielded the same results (Figure 1E). Thus, the effect is at the protein level and not at the level of transcription. We conclude that PtCAF1 and Ezl1 are mutually dependent on one another, possibly at the level of protein stability.

Another explanation of their high correlation when silenced is that they are parts of the same complex, which we deemed the most likely scenario because PtCAF1 and Ezl1 could both be subunits of a PRC2-like complex. To test the interaction between Ezl1 and PtCAF1, we co-expressed constructs of the genes with Myc and FLAG-hemagglutinin (HA) tags and performed immunoprecipitation (IP) using an anti-HA antibody. Due to their dual localization pattern in both early and late development, samples were collected from both stages. In both cases and in both developmental time points, the partner co-precipitated with the bait and could be detected by western blot with the anti-Myc antibody (Figure 1B). The interaction could be robustly detected in native conditions (without crosslinking), which suggests a strong interaction between the proteins. Taken together, our results demonstrate an interaction of Ezl1 and PtCAF1 and show that they are mutually dependent on each other for their stability.

### Identification of the core components of PRC2

The observation that Ezl1 and PtCAF1 interact suggests that they may be a part of a PRC2-like complex. PcG proteins generally assemble into one of two multiprotein complexes that post-translationally modify histones: PRC1, which mono-ubiquitylates histone H2A at lysine 119 (H2AK119ub1); and PRC2, which trimethylates lysine 27 of histone H3 (H3K27me3) (de Napoles et al., 2004; Wang et al., 2004; Cao et al., 2002; Czermin et al., 2002; Kuzmichev et al., 2002; Müller et al., 2002). To characterize the remaining members of the *Paramecium* PRC2 complex, we performed IP using PtCAF1 as a bait that was followed by protein mass spectrometry, as well as IP and mass spectrometry of WT cells as a negative control (Figures S2A–S2E). Because the PtCAF1-Ezl1 complex is expressed in both early and late stages of development, we collected samples from both time points. Mass spectrometry identified the following PtCAF1-associated proteins: Ezl1, Eed, Suz12, Rnf1, and Rnf2 (Figures S2D and S2E; Table S1). The complex was further validated by IP and subsequent mass spectrometry of two of the PtCAF1-associated proteins, Eed-FLAG-HA and Ezl1-FLAG-HA, and all the same subunits were co-precipitated in these IPs as well (Figures S2F–S2I; Table S1).

Eed has a WD40 repeat domain, which is also found in other organisms (Figure 1D). Suz12 has a putative Zinc-finger and VEFS domain (domain found in the the C-terminal region of the VRN2, EMF2, FIS2, and Su(z)12 polycomb proteins). In addition, it is considerably shorter than the canonical Suz12 found in other organisms (291 amino acids [aa] versus more than 700 aa in human, mouse, and fruit fly) due to the absence of most of the N-terminal part. Both Rnf1 and Rnf2 have Zinc-finger, coiled-coil, and Ring domains. All six genes show a similar expression pattern, and their mRNAs are expressed during the early developmental stage (Figure 1C). Although the protein complex is present in two distinct nuclei, the composition of the complex appears to be the same in both early and late development (in the maternal MAC and the newly developing MAC). To verify the interactions, we focused on proteins that comprise the minimal PRC2 complex required for enzymatic activity: Ezl1, Eed, and Suz12 (Cao and Zhang, 2004). Although *Paramecium* Suz12 is shorter than in most organisms, it does contain the VEFS domain, which was shown to be necessary to stimulate the methyltransferase activity of EZH2. Because we used PtCAF1 as the bait for the mass spectrometry, we proceeded to test the interaction between the minimal PRC2 complex and PtCAF1. Eed-Myc and Suz12-Myc were each co-expressed with a FLAG-HA-tagged PtCAF1, and IP was performed as described above. In both cases, the partner co-precipitated with the bait, confirming the interactions of the minimal PRC2 complex and PtCAF1 (Figure 1B). Both proteins were also tagged with GFP, and their localization was followed throughout development. Eed-GFP and Suz12-GFP localized similar to PtCAF1-GFP, with a dual-localization pattern and foci formation in the new MACs (Figures S3A and S3B).

The two remaining subunits, Rnf1 and Rnf2, contain conserved Ring-finger domains and are not standard PRC2 complex subunits. These proteins resemble Ring-finger domain proteins normally found in the PRC1 complex, responsible for mono-ubiquitylating histone H2A at lysine 119 (H2AK119ub1). It is therefore tempting to speculate that there might exist a fusion of the PRC1 and PRC2 complexes in *Paramecium*. We therefore tested this hypothesis by immunofluorescence of H2AK119ub1 during development and its dependence on two PRC2 subunits: Ezl1 and Rnf1. Although H2AK119ub1 is indeed present at the same time points and nuclei as the PRC2 complex, we observed no dependence on either Ezl1 or Rnf1 (Figure S4). We conclude that the core PRC2 is not required for H2AK119ub1, nor is the accessory subunit Rnf1. Next, we expressed GFP fusions of both proteins to determine their sub-cellular localizations. Rnf2-GFP showed the same dynamic localization pattern as the core PRC2 complex (Figure S3C). In contrast, Rnf1-GFP was found in the cellular cortex and maternal MAC in very early stages but was present for only a brief period of time in the new MACs in later stages of development (Figure S3D). Unlike the rest of the subunits, Rnf1-KD does not alter the localization of PtCAF1-GFP, Ezl1-GFP, or Rnf2-GFP in the new MACs (Figures 1E–1G). Similarly, none of the core-PRC2 subunits alter the localization of Rnf1 (Figure 1H). Hence Rnf1 displays a different localization to all the other subunits and might serve as an accessory protein mainly for its function in the maternal MAC. Its role in the new MACs, if any, remains to be determined.

Because PtCAF1 and Ezl1 are required for H3K9me3 and H3K27me3 deposition during sexual development, we performed immunofluorescence after depletion of each of the PRC2 subunits we identified. Silencing of Ezl1, PtCAF1, Suz12, Eed, or Rnf2 all abolish the methylation marks in both nuclei (Figure 1I). Contrary to this, the depletion of Rnf1 does not affect the new MAC localization of either methylation mark, but the H3K27me3 staining in the maternal MAC was abolished, further supporting our hypothesis that Rnf1 is mainly involved in the maternal MAC. Taken together, we conclude that the PRC complex in *Paramecium* is a prototypical PRC2 complex in both nuclei required for the deposition of H3K9me3 and H3K27me3, and it consists of the core components of Ezl1, PtCAF1, Suz12, Eed, and Rnf2, as well as the accessory subunit Rnf1 (Figure 1J).

### The PRC2 complex is required for DNA elimination during development

**Figure 2:**
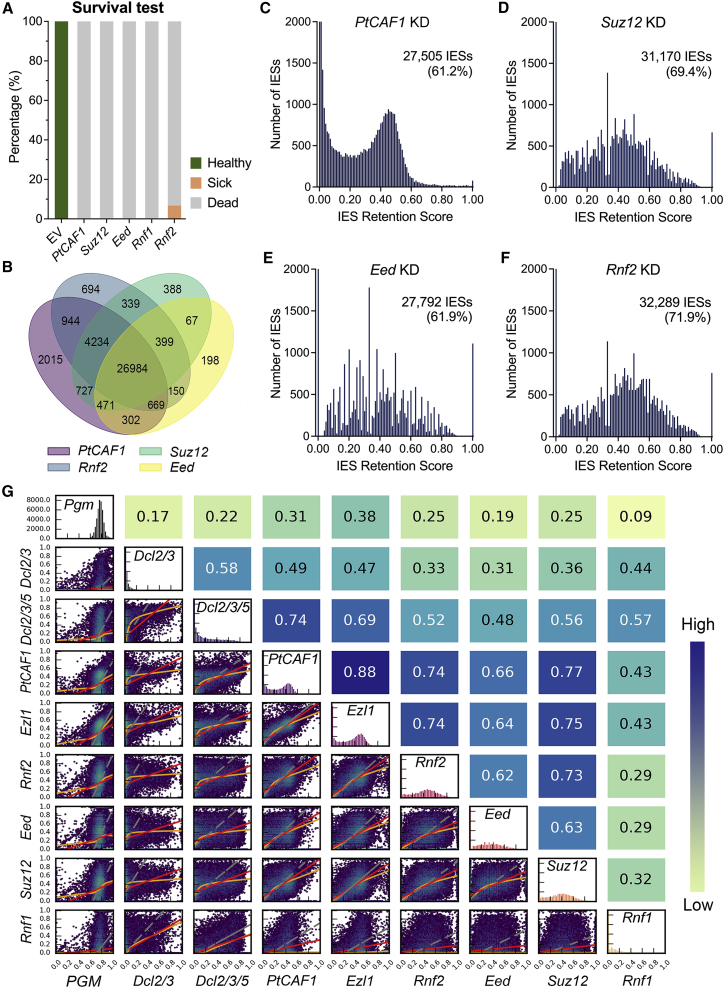
The PRC2 complex is required for DNA elimination during development (A) Survival test after silencing, n = 30. n is the number of cells examined. Green: healthy; pink: sick; gray: dead. (B) Venn diagram depicting shared IES retention between *Suz12*, *Eed*, *Rnf2*, and *PtCAF1* KD. (C–F) IES retention scores (IRSs) after *PtCAF1* (C), *Suz12* (D), *Eed* (E), and *Rnf2* (F) KD. Numbers under the gene names show the number and percentage of IESs with IRS ≥ 0.1. (G) Correlation plots of KDs calculated by hexagonal binning of IRS. Pearson’s correlation coefficients are given above each subgraph. Red lines are for ordinary least-squares (OLS) regression, orange lines for locally weighted scatterplot smoothing (LOWESS), and gray lines for orthogonal distance regression (ODR). From light green to dark blue, the correlation is stronger. See also Figure S5.

To assess the contribution of the individual components of the complex, we silenced the newly identified subunits during development. To test their survival rate, we re-fed the cells and allowed them to multiply. Depletion of any PRC2 subunit leads to a complete inability to resume vegetative growth (Figure 2A). To obtain a more comprehensive view of the effects on DNA elimination events, we sequenced the new MAC genomes after silencing and compared them with the previously published PtCAF1-KD and Ezl1-KD new MAC genomes (Ignarski et al., 2014; Lhuillier-Akakpo et al., 2014). Depletion of any PRC2 core component mimicked PtCAF1-KD, indicating that each subunit is likely essential for the function or stability of the complex (Figures 2B–2F). Each silencing leads to the retention of 60%–70% of all IESs (IES retention score [IRS] ≥ 0.1), with both long and short IESs being affected (Figures 2C–2F and S5A–S5E). The depletion of the accessory subunit, Rnf1, has a very different effect on DNA elimination: much fewer IESs are retained, and the average retention score is very low (Figures S5F and S5G). In total, 20.4% of IESs (IRS ≥0.1) were retained, nearly all of which fall within the PtCAF1-KD affected subset (Figure S5H). Furthermore, the IESs affected by Rnf1-KD are mainly sRNA dependent (Dcl2/3/5 dependent), although not all sRNA-dependent IESs require Rnf1 (Figure S5H). In summary, PRC2 is involved in the elimination of transposons and transposon-derived sequences (IESs) and is essential for development. Moreover, the core components of PRC2 are essential for its activity, because neither of them is dispensable. Although the accessory subunit, Rnf1, may well modulate PRC2 activity or at least part of its functions, most DNA elimination events can occur in its absence, and it displays a different localization to the rest of the complex. However, Rnf1 is required for post-developmental cell survival, as well as transposon and IES elimination, yet its exact function remains to be elucidated.

### Loss of PRC2 impairs the RNA scanning process

**Figure 3:**
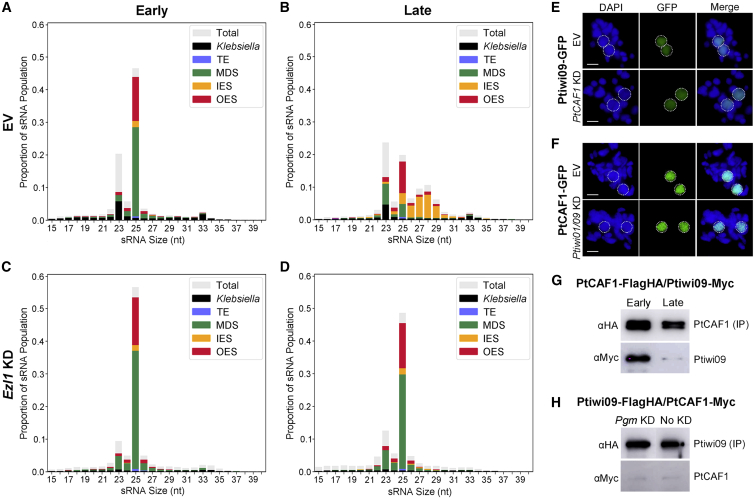
Loss of PRC2 impairs the RNA scanning process and PRC2 interacts with Ptiwi09 (A–D) sRNAs of EV and *Ezl1* KD in both early and late time points. (E) Ptiwi09-GFP localization in the late stage on EV and *PtCAF1* KD. (F) PtCAF1-GFP localization in the late stage on EV and *Ptiwi01/09* KD. (G) Western blot of HA-immunoprecipitated proteins using anti-HA or anti-Myc antibodies to detect PtCAF1-FLAG-HA and Ptiwi09-Myc. (H) Western blot of HA-immunoprecipitated proteins using anti-HA or anti-Myc antibodies to detect Ptiwi09-FLAG-HA and PtCAF1-Myc on *Pgm* KD and without KD. Samples are from the late time point. Scale bars: 10 μm. EV, empty vector silencing; IES, internally eliminated sequence; MDS, macronuclear destined sequence (MAC matching); no KD, no silencing; OES, other eliminated sequence; TE, transposable element. See also Figure S6.

As previously mentioned, the PRC2 complex appears to be active both in the maternal MAC in early stages and in new MACs in later stages. We therefore first investigated its role in the maternal MAC. Total RNA was extracted from a culture depleted of Ezl1, from both early and late time points, and sRNAs were sequenced and quantified. The amount of 25-nt scnRNAs in the early time points is comparable between the control and Ezl1-KD, and there was no noticeable negative effect on scnRNA production (Figures 3A and 3C). In the late time point of the control, there is a decrease in MAC-matching compared with IES-matching scnRNAs, the expected consequence of the scanning process (Figure 3B). The appearance of iesRNAs in the late time point is also visible by the exclusively IES-matching class of 26- to 31-nt sRNAs (Figure 3B) (Sandoval et al., 2014). Comparatively, in the late time point of Ezl1-KD, the ratio of MAC-matching (somatic) to IES-matching (germline-limited) scnRNAs is nearly unchanged compared with the early time point (Figure 3D). In addition, there are no iesRNAs, a feature that may be attributed to a lack of IES excision, a pre-requisite for circularization and iesRNA production (Allen et al., 2017). Because the accessory subunit, Rnf1, displayed a different IES retention pattern to the rest of the complex, we also investigated the effect of its depletion on sRNAs. A near-identical effect on scanning and a block in the iesRNA pathway were also observed for this subunit (Figure S6). This is surprising, because a complete lack of iesRNAs in the absence of Rnf1 cannot be attributed to a lack of IES excision, because its effect on IESs is only moderate. Taken together, the PRC2 complex is indispensable for the scanning process but does not affect scnRNA biogenesis. In addition, its depletion leads to a block in the iesRNA pathway.

### PRC2 interacts with Ptiwi09 and couples Piwi-bound sRNAs to heterochromatin formation of transposons

We and others have shown H3K9me3 and H3K27me3 to be scnRNA dependent (Ignarski et al., 2014; Lhuillier-Akakpo et al., 2014). It is therefore possible that the above-described effect on scnRNAs is due to retention of scnRNA-Ptiwi complexes in the maternal MAC on PRC2 depletion. To test this, we expressed Ptiwi09-GFP in a PtCAF1-KD background and observed throughout autogamy. Ptiwi09 was able to localize normally to both nuclei, with no observable retention in the maternal MAC in later stages (Figure 3E). Thus, PRC2 is not involved in the trans-nuclear transport of Ptiwi09, nor does its depletion sequester non-scanned Ptiwi09 in the maternal MAC. To test the opposite, we performed the reciprocal experiment by Ptiwi01/09 silencing in cells expressing PtCAF1-GFP. Also in this case, the localization of PtCAF1 was unchanged (Figure 3F). We conclude that neither protein is dependent on the other for their trans-nuclear transport.

The protein mass spectrometry results of PtCAF1 shed light on the unexplained link between PRC2 and Ptiwi09: in both time points, Ptiwi09 could be identified (Table S1). The interaction was confirmed in both time points and appears to be stronger in the early time point (Figures 3G and 3H). Because we performed these experiments in native conditions, it is possible that transient or weak interactions are lost because only the strongest interactions can be maintained by our approach. To arrest the complex at a stage where DNA has not yet been eliminated, we tested the interaction on Pgm-KD, which blocks the final step of DNA elimination. Nonetheless, the interaction did not get stronger, suggesting that DNA elimination is not what disrupts their interaction (Figure 3H). Although the complex appears to be present only in the new MACs in later stages, we cannot exclude that a part of the complex is retained in the maternal MAC in the time points we collected. It is therefore possible that the interaction we detect is from the maternal MAC and not the new MACs, despite Ptiwi09 being consistently among the top hits in the mass spectrometry results also in the late stage. We conclude that PRC2 and Ptiwi09 interact, and that this interaction is upstream of DNA elimination and is likely transient.

### The involvement of PRC2 in DNA elimination is mainly fulfilled by the new MAC complex

**Figure 4:**
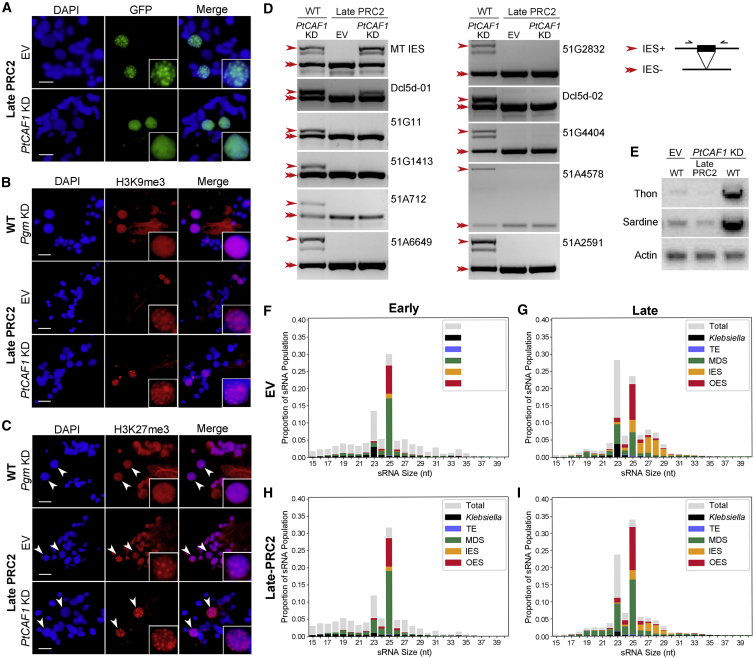
The involvement of PRC2 in DNA elimination is mainly fulfilled by the new MAC complex (A) Localization of recodonized PtCAF1-GFP in cells co-expressing Ezl1, Suz12, Eed, and Rnf2 on EV and endogenous *PtCAF1* KD. (B and C) Immunofluorescence of H3K9me3 (B) and H3K27me3 (C) in late PRC2-expressing cells on EV and endogenous *PtCAF1* KD, as well as *PGM* KD in WT cells. Arrows point to new MACs. (D) IES retention PCR in WT and late-PRC2 expressing cells, with and without *PtCAF1* KD. Single arrows indicate bands with the IES, and double arrows indicate bands without the IES. (E) Transposon retention in new MACs. Thon and Sardine are two classes of transposons. Actin is the loading control. (F–I) sRNA population of late-PRC2-expressing cells in EV and *PtCAF1* KD. Scale bars: 10 μm. EV, empty vector silencing; IES, internally eliminated sequence; late PRC2, new MAC-specific PRC2-expressing cells; MDS, macronuclear destined sequence (MAC matching); OES, other eliminated sequence; TE, transposable element; WT, wild type. See also Figure S7.

The localization of the PRC2 complex, as well as its importance for scanning in early stages, suggests dual functions: one in the maternal MAC and one in the new MACs. As shown above, the PRC2 complex localizes to the maternal MAC and is necessary for scanning. To establish whether IES retention is solely a result of the role of PRC2 in the maternal MAC or if it also has a role in the late stage, we sought to isolate its function in the new MACs. This is challenging, because the effect in the maternal MAC can mask the new MAC-related phenotype, making it impossible to directly assign the effects to one or the other when using RNAi as a tool. Taking advantage of the newly identified PRC2 subunits combined with destabilizing the endogenous complex allowed us to tackle this question. First, we recodonized PtCAF1 to make it resistant to silencing of the endogenous PtCAF1 (Table S2). When the recodonized PtCAF1 is expressed under its endogenous promoter, the protein localizes normally and can rescue the silencing of the endogenous PtCAF1, displaying no lethality or IES retention after autogamy (Figures S7A–S7C). Hence the recodonized PtCAF1 is functional. Next, the endogenous complex was removed by PtCAF1-KD, having previously shown that the complex is not stable without PtCAF1, and a combination of RNAi-resistant PtCAF1-GFP, as well as Ezl1, Eed, Suz12, and Rnf2, was introduced under the control of the Ku80c promoter. This promoter was chosen based on a previous study of the Ku80c protein and a RNA sequencing (RNA-seq) dataset in which it displays a strong expression exclusively late in development (Figure S7E) (Abello et al., 2020; Arnaiz et al., 2020). This allowed us to express the complex exclusively in new MACs to assess its role in late development. For clarity, we use the term “late-PRC2” to refer to the complex when it is exclusively expressed in the late stage. The mix was first introduced in control cells, without PtCAF1-KD, to ascertain that the complex is correctly expressed and localized when delayed. We observed no dominant-negative effects by introducing the complex (Figures 4D and S7C). The late-PRC2 enters the new MACs specifically, with no observable signal in the maternal MAC, and forms the same foci as when expressed under its own promoter (Figure S7D). The exact nature of these foci in *Paramecium* is unknown; however, PcG proteins are well known to organize into nuclear domains known as “Polycomb bodies” or “Polycomb foci,” suggested to be a nexus for Polycomb target sites via chromatin looping (Entrevan et al., 2016; Cheutin and Cavalli, 2014; Mao et al., 2011). The presence of Polycomb bodies might indicate that the targets of PRC2 cluster together in the 3D space of the nucleus; however, further experiments are required to determine if this is the case.

Next, PtCAF1 was silenced in the transformed cells, as well as in WT as a control. The late-PRC2 localizes to the new MACs; however, foci formation is abolished (Figure 4A). This phenotype is reminiscent of Ezl1 localization on Pgm-KD, after which the foci in new MACs are unable to form, yet the methylation marks are present and also dispersed (Figures 4B and 4C) (Lhuillier-Akakpo et al., 2014). In the case of Pgm-KD, all IESs and transposons are retained, and it can therefore be speculated that the reason for dispersion of the foci is a consequence of DNA not being removed, and thus there is an accumulation of methylation marks on sequences destined for elimination as development progresses. In the case of late-PRC2, one can imagine at least two explanations for the dispersion of the foci: (1) no targeting by scnRNAs, or (2) too many targets of scnRNAs. To tackle this question, we investigated the localization of H3K9me3 and H3K27me3 by immunofluorescence staining. In addition to the negative control, Pgm was also silenced in WT cells, and we observed the methylation marks to visualize what it looks like without foci. Both H3K9me3 and H3K27me3 formed foci resembling the control when PtCAF1 was silenced, whereas the foci were completely abolished on Pgm-KD (Figures 4B and 4C). It appears that the late-PRC2 is still able to set methylation marks in the new MACs, which no longer co-localize with the complex itself. To determine whether DNA elimination is also rescued, we extracted genomic DNA after completion of autogamy and performed IES retention PCRs. Most of the IESs assayed were correctly excised: 9 of the 11 IESs in the PRC2-dependent subset could be rescued by late-PRC2 expression (Figure 4D). The retention of TEs was also tested by PCR. In contrast with PtCAF1-KD in WT cells, in which the TEs are retained, late-PRC2 expression can rescue TE elimination (Figure 4E). To get a better overview of the rescue, whole-genome sequencing was performed. Indeed, the IES retention pattern is left shifted, displaying a much weaker effect on IESs (Figure S7F). Nonetheless, cells are unable to return to vegetative growth and gradually die after re-feeding (Figure S7C).

The discrepancy between the localization of PRC2 and the methylation marks prompted us to investigate more directly if the dispersion of PRC2 could be caused by an overabundance of scnRNAs in the new MACs. To test this, we performed the same experiment by expressing late-PRC2, but this time Dcl2/3 was co-silenced with PtCAF1. Because Dcl2/3 cooperate to produce the scnRNAs, if the disruption of the foci is caused by an overabundance of scnRNAs, this should rescue the phenotype (Sandoval et al., 2014; Lepere et al., 2009). Indeed, the foci of PRC2 were rescued on Dcl2/3-KD, yet their localization appears altered compared with the non-silenced control (Figure S7G). This may suggest that the dispersion is caused by an overabundance of scnRNAs. To examine the sRNA population, we extracted total RNA from late-PRC2 transformed cells in control and PtCAF1 silencing, and sRNAs were sequenced and quantified. Late-PRC2 expression rescued the iesRNA population, but only partly rescued the scanning defect (Figures 4F–4I). Taken together, our results demonstrate that PRC2 is essential for DNA elimination mainly through its function in the new MACs, and that its role in early stages is dispensable for most DNA elimination events. These results imply that non-scanned scnRNA-Ptiwi complexes enter new MACs and correctly guide PRC2 to most IESs and TEs. The dispersion of the Polycomb bodies may therefore be a consequence of too many targets, not a lack of chromatin association.

### *Paramecium* PRC2 does not appear to perform the classical function of PRC2 complexes

**Figure 5:**
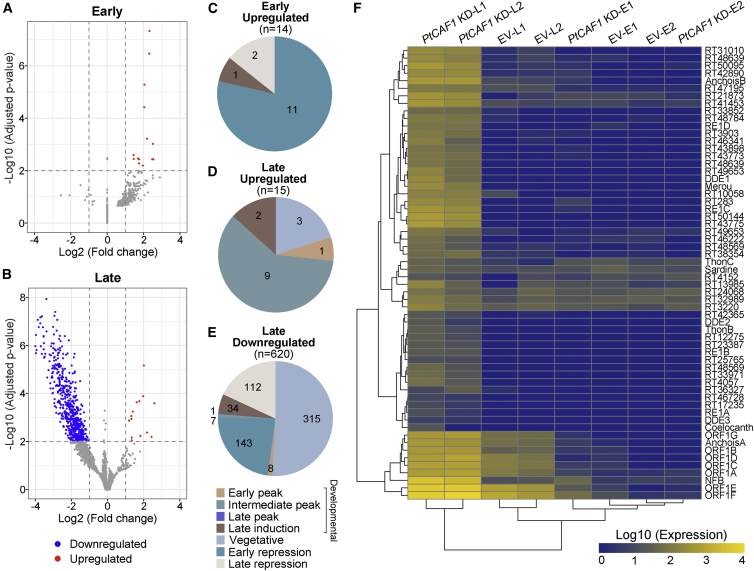
PRC2 does not appear to perform the classical function of PRC2 complexes (A and B) Volcano plots of differentially expressed genes (DEGs) between *PtCAF1* KD and EV in early (A) and late (B) time points. Genes with fold change ≥ 2 and adjusted p < 0.01 are classified as DEGs. Red dots indicate upregulated genes, and blue dots represent downregulated genes. (C–E) RNA-seq expression classifications of DEGs with at least a 2-fold change compared with EV. n is the number of DEGs. (F) Heatmap of transposon expression in EV and *PtCAF1* KD. Each row represents a predicted transposon. Blue to yellow colors represent the log10 of calculated expression values. Letters “E” and “L” indicate early and late time point. Numbers “1” and “2” are replicates. EV, empty vector silencing. See also Table S3.

The catalytic subunit of the PRC2 complex, Ezl1, has recently been shown to possess a dual-methyltransferase activity, methylating both H3K9me3 and H3K27me3 (Frapporti et al., 2019). In higher eukaryotes, H3K27me3 is considered a hallmark of PRC2-mediated gene repression. Because H3K27me3 is set by PRC2 in the maternal MAC during early development, it is possible that the effects of silencing on DNA elimination are due to gene expression changes. To investigate whether there are changes in the transcriptome on PRC2 depletion, we sequenced and quantified total mRNAs from early and late time points of control and PtCAF1-KD. In the early time point, only 14 mRNAs showed a change in the expression level compared with the control (Figure 5A). All 14 showed an upregulation of expression (Figures 5A and 5C; Table S3). In the late time point, 635 genes were differentially expressed, with 98% (620) of them downregulated (Figures 5B, 5D, and 5E; Table S3). Because the classical function of PRC2 complexes is gene repression, we would expect an upregulation in its absence. Instead, we observed the opposite, with only a handful of upregulated genes in each time point (Figures 5A–5E). We conclude that the depletion of PRC2 does not lead to a global upregulation of transcription. Interestingly, many genes were downregulated in the late time point (Figure 5B). We then investigated the differentially expressed genes by examining their expression profiles using published RNA-seq datasets and their classifications (Arnaiz et al., 2020). Most of the genes affected by PRC2-KD are expressed during vegetative binary fissions, and only 50 are classified as development specific (Figures 5C–5E). Although this suggests a vegetative function rather than a developmental one, an indirect effect on scanning or DNA elimination events cannot be excluded. Moreover, we observed a significant upregulation of transcripts mapping to TEs, indicating that PRC2 is required for preventing TE expression (Figure 5F). Taken together, our RNA-seq data exclude a global upregulation of transcription in the absence of PRC2, and its role in the maternal MAC is unlikely to be the classical PRC2 function of gene repression. The de-repression of TEs observable in our dataset rather suggests that PRC2 is required for controlling TEs both at the level of transcription and DNA elimination. Whether the PRC2 complex has a function in activating transcription needs further investigation.

### Heterochromatin formation drives the elimination of TEs, but not IESs

TEs in higher eukaryotes are strictly controlled by repressive chromatin marks such as H3K9me3 and heterochromatin formation to ensure a silent state. In ciliates, TEs are present only in the transcriptionally inactive germline during vegetative growth; consequently, they are unable to replicate. During the formation of a new soma, however, TEs need to be strictly controlled during the sudden burst of transcription and massive genome rearrangements that occur, until they can be eliminated for good. Recently, reports of global de-repression of TEs in the absence of Ezl1 were reported in both *Tetrahymena* and *Paramecium*, highlighting its role in maintaining TEs in a silent state (Frapporti et al., 2019; Zhao et al., 2019). In *Tetrahymena*, ChIP-seq data revealed that H3K9me3 and H3K27me3 accumulate at both IESs and TEs, although this was shown only to decorate the latter in *Paramecium* and was not analyzed for IESs likely because of low coverage of the sequencing (Frapporti et al., 2019). Therefore, the role of these methylation marks and heterochromatin formation on IES elimination remains unclear.

**Figure 6:**
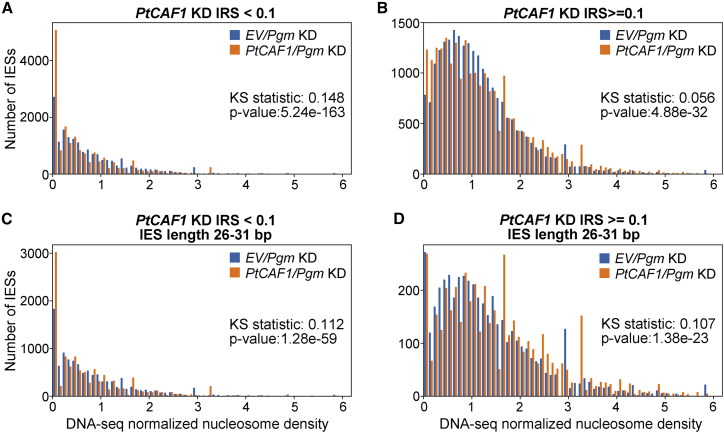
PRC2 affects the nucleosomal landscape of IESs Normalized nucleosome density histograms for IESs weakly and strongly affected by *PtCAF1* KD. Histograms for the scores r_e_ (EV/PGM-KD) and r_p_ (PtCAF1/PGM-KD). (A and B) Subplots for all IESs with the given *PtCAF1* KD IES retention score cutoff. (C and D) Subplots have the additional constraint of being limited to the first IES length peak (26–31 bp). KS statistics are for two sample tests with a two-sided alternative hypothesis.

To approach this question more directly, we performed nucleosome profiling in PtCAF1-silenced cells to determine whether there are changes to nucleosome densities on IESs when PRC2 is absent. In both the control and PtCAF1-KD, Pgm was co-silenced to retain all IESs for the analyses. Nucleosome densities were lower for IESs weakly affected by PtCAF1-KD (IRS < 0.1), irrespective of the KD (Figures 6A and 6B; in all cases with a Kolmogorov–Smirnov (KS) two-sided test, alpha = 0.05, the hypothesis that the distributions are equal would be ruled out). Differences between PtCAF1/Pgm-KD and EV/Pgm-KD nucleosome density distributions were much more subtle than the pronounced differences with respect to IRS threshold, e.g., the proportion of IESs with zero or close to zero nucleosomal densities in the lowest histogram bin are higher in PtCAF1/PGM-KD. Because PtCAF1-KD more strongly affects longer IESs, we compared nucleosome densities for IESs of the same lengths (26–31 bp), corresponding to the first peak of the IES length distribution. For IRS < 0.1, the distributions of nucleosome densities of this subset paralleled the IES population as a whole, but not for IRS ≥ 0.1 (Figures 6C and 6D). Overall, the nucleosome densities on IESs appear altered by PtCAF1-KD, but more notable is the greater association of higher nucleosome densities with stronger PtCAF1-KD effect on IES excision. Hence the alterations in nucleosome densities caused by silencing of PtCAF1, a PRC2 subunit, are not a simple decrease for PtCAF1-sensitive IESs as one might expect if the IESs were heterochromatinized in a PRC2-dependent manner. For PtCAF1-sensitive IESs, both a greater proportion of IESs have lower (e.g., <0.5) and higher (e.g., >2) nucleosome densities for PtCAF1/Pgm-KD than EV/Pgm-KD (for all IESs; Figure 6B), whereas for length peak 1 IESs, a greater proportion of IESs have higher nucleosome densities in PtCAF1/Pgm-KD (Figure 6D).

**Figure 7:**
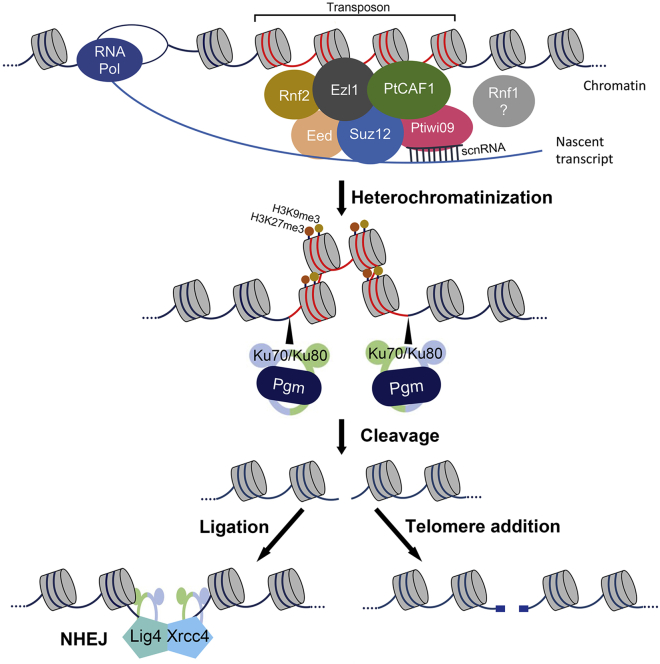
Proposed model of PRC2 in transposon elimination scnRNA-Ptiwi09 complexes target transposon transcripts and guide the PRC2 complex to transposon sequences. Ezl1 catalyzes H3K9me3 and H3K27me3, resulting in heterochromatinization and transcriptional repression. The domesticated transposase Pgm cleaves the transposon sequence, after which the double-stranded break is either repaired by NHEJ with the help of Ligase IV and Xrcc4, or telomeres are added *de novo* to the ends. Note that the interactions between the subunits depicted in the schematic do not represent precise interactions.

Considering that 93% of IESs in *Paramecium* are shorter than the size of a nucleosome and are dispersed throughout the genome, it seems unlikely that heterochromatin formation can play a major role in the elimination of IESs. This conclusion is further corroborated by findings that IESs appear to be nucleosome poor (Singh et al., 2022). Taken together, this suggests that precise and imprecise DNA elimination events must follow two distinct pathways, the former of which is mediated through nucleosome depletion and the latter through heterochromatin formation. We propose the following model for PRC2-mediated elimination of TEs (Figure 7): scnRNA-Ptiwi09 complexes guide the PRC2 complex to transposons in a sequence-specific manner. The catalytic subunit, Ezl1, deposits H3K9me3 and H3K27me3, which leads to heterochromatin formation and prevents their expression. The domesticated piggyBac transposase, Pgm, as well as the Ku70/80c heterodimer, are recruited and remove the transposon sequences (Marmignon et al., 2014; Baudry et al., 2009). Lastly, Xrcc4 and Ligase IV repair the double-stranded breaks by non-homologous end joining (NHEJ), or telomeres are added *de novo* to the ends (Kapusta et al., 2011).

## Discussion

PRCs and the piRNA pathway are generally considered functionally distinct. This notion is challenged by our results that a PRC2 complex in *Paramecium* couples Piwi-bound sRNAs to heterochromatin formation of transposon sequences, ultimately leading to the elimination of their DNA. This may in part be explained by the dual-methyltransferase activities of the Ezl1 protein, capable of performing both H3K9me3 and H3K27me3 (Frapporti et al., 2019). Consequently, this enables the protein to perform the activities of the SETDB1 family and the EZH family of methyltransferases. Regardless, we found no indication of the classical function of PRC2 (i.e., gene repression) of the *Paramecium* complex; thus, its main function appears to be limited to excision of transposons and transposon-derived DNA.

### The *Paramecium* PRC2 complex

To investigate the elusive Ezl1 complex in *Paramecium*, we performed IP experiments to characterize its components and identified a prototypical PRC2 complex (Figure 1). Although the complex contains the conserved Ezl1 (EZH1/2), Suz12 (SUZ12), Eed (EED1), and PtCAF1 (RbAp46/48) subunits, it also contains two Ring-finger domain proteins (Rnf1 and Rnf2). At the time of this study, no PRC complex had been characterized in any ciliate. However, recently, a PRC-like complex was reported in a distantly related ciliate, *Tetrahymena thermophila* (Xu et al., 2021). As we have shown in *Paramecium*, the *Tetrahymena* complex also consists of EZL1 (EZH1/2), SUZ12 (SUZ12), ESC1 (EED), RNF1, and RNF2. Interestingly, no RbAp46/48 homolog co-purified with the complex, and the authors argue that the interaction interface between a putative RbAp46/48 and SUZ12 is not conserved, because SUZ12 lacks the N-terminal extension required for such an interaction. In contrast with this hypothesis, Suz12 in *Paramecium* also lacks the N-terminal extension, yet it could robustly pull down a RbAp46/48 homolog in our screens, PtCAF1 (Figure 1B). It is therefore plausible that the interaction of Suz12 and PtCAF1 occurs through an alternative interface, or their interaction may be indirect. Similar to the case in *Paramecium*, *Tetrahymena* PRC also contains Ring-finger domain proteins (RNF1 and RNF2), suggesting that this may be a common feature of PRC complexes in ciliates. During the revision of this manuscript, the PRC2 complex in *Paramecium* was reported in a separate study (Miró-Pina et al., 2022). This study complements our own, and both provide different lines of evidence for the presence of this complex. First, the same subunits were found, but both studies investigated only a subset in detail. This includes the identification of four core components, PtCAF1 (PtCAF1), Ezl1 (Ezl1), Eed (Eed), and Suz12 (Suz12.like), strengthening the claim of the complex. Our study provided *in vivo* evidence to demonstrate the requirement of each subunit for the stability of the complex, as well as coimmunoprecipitation (coIP) to confirm the interactions, while the other study demonstrated this *in vitro* using a heterologous insect system. By focusing on the subunits that compose the core PRC2 in other eukaryotes, the other study failed to identify Rnf2 (Rf2) as a core subunit, while our *in vivo* study provided evidence that Rnf2 is indeed a core PRC2 subunit in *Paramecium*. This may also explain the lack of methyltransferase activity of the complex composed only of PtCAF1, Ezl1, Eed, and Suz12, suggesting that Rnf2 may be required for the activity of the complex. Second, the interaction with Ptiwi09 was found in both studies. Following up on this, the other study demonstrated that this interaction is independent of nucleic acids, suggesting a direct interaction, and that the RF4 subunit (Rnf1 in our study) bridges the interaction between the PRC2 complex and Ptiwi09. Furthermore, our study used a combination of a new MAC-specific PRC2 complex, RNA-seq analysis, and nucleosome profiling to separate the functions in the maternal and new MACs, demonstrating that the PRC2 complex is mainly required for DNA elimination in the new MACs and suggesting that most IESs are likely not heterochromatinized in a PRC2-dependent manner in *Paramecium*.

### A putative role of PRC2 in the maternal MAC

The scanning model posits that scnRNAs matching somatic sequences are removed and only the scnRNAs matching sequences that were not present in the soma of the previous generation are transported to the new MACs (Mochizuki and Gorovsky, 2004; Mochizuki et al., 2002). However, the exact mechanism of degrading MAC-matching scnRNAs is unknown. We have shown that PRC2 is required for degradation of MAC-matching scnRNAs, yet its depletion does not retain the scnRNA-binding Ptiwi09 in the maternal MAC. This suggests that MAC-matching scnRNAs may be transported to the new MACs on PRC2 depletion, contrary to the model. Moreover, a new MAC-specific PRC2 complex could rescue most DNA elimination events, including elimination of TEs, as well as restore iesRNAs and to some extent MAC-matching scnRNA degradation, and its role in the maternal MAC thus appears at least partly dispensable for these processes (Figure 4). We propose that the role of PRC2 in the maternal MAC is to ensure the degradation of scnRNAs matching somatic sequences, yet its precise role in this process is still unknown.

### Imprecise DNA elimination is governed by a sRNA-guided PRC2 complex

Although both TEs and IESs are affected by PRC2 depletion, TEs are the only ones shown to be enriched in the methylation mark PRC2 sets (Frapporti et al., 2019). Rather exceptionally, Ezl1 in *Paramecium* was shown to possess dual-methyltransferase activities, by trimethylating both lysine 9 and lysine 27 of histone H3 (Frapporti et al., 2019). Although these marks are found at different elements and are set by different enzymes in other organisms, in ciliates they appear to decorate the same DNA sequences destined for elimination. Several lines of evidence have demonstrated that these methylation marks are sRNA dependent, yet an indirect effect has not been excluded. Our RNA-seq data on PRC2 depletion do not support an effect on gene repression; however, most of the affected genes were downregulated in its absence (Figure 5). Therefore, we cannot exclude the possibility that *Paramecium* PRC2 is involved in transcriptional activation. Although the classical function of PRC2 complexes is gene repression, there have been reports that EZH2 can function as a transcriptional activator in a PRC2-independent manner (Kim et al., 2015, 2018; Xu et al., 2012). A recent report found the vegetative chromatin landscape of *Paramecium* to be different from most eukaryotes, and that H3K27me3 accumulates on highly expressed genes, questioning its function as a repressive mark in this system (Drews et al., 2022). Whether *Paramecium* PRC2 is indeed a transcriptional activator, or if it is an indirect effect, remains to be determined. We also demonstrated the importance of PRC2 in both transcriptional repression of TEs and their removal (Figures 5 and 7). Complementing these observations, our results showed that the PRC2 complex interacts with Ptiwi09, which suggests a more direct involvement than previously anticipated (Figures 3G and 3H). However, the implications of this interaction remain to be unveiled.

There are striking similarities of the TE elimination in ciliates to the mechanisms by which TEs are repressed in animal germlines. They both appear to require (1) Piwi proteins and sRNAs, (2) methyltransferase setting repressive methylation marks, and (3) formation of heterochromatin. Despite the ultimate outcome of DNA elimination in ciliates, the same players are involved in the same order, up until that point. Accordingly, the mechanism of controlling TEs in ciliates is functionally analogous to the piRNA pathway in animal germlines.

### PRC2 is involved in removal of DNA sequences shorter than a nucleosome

It has long been known that heterochromatin formation forms the essence of DNA elimination in *Tetrahymena* (Noto and Mochizuki, 2017; Chalker et al., 2013). Until recently, a direct involvement of such factors was deemed unlikely in *Paramecium*, and an indirect effect on transcription could not be excluded. After all, there are striking differences between the characteristics of IESs in *Tetrahymena* and in *Paramecium*, the most significant of which are the length and the imprecise nature of IES elimination in *Tetrahymena* (Hamilton et al., 2016). Meanwhile, IESs in *Paramecium* are located within coding regions and are thus subject to constraints of precise elimination to form functional genes (Arnaiz et al., 2012).

Somewhat counterintuitively, our data highlight the vital role PRC2 also plays in the removal of extremely short DNA sequences, the IESs, an effect unlikely to be attributed to gene expression changes. To reconcile these seemingly contradictory observations, we postulate that the elimination of new and ancient transposons enters two different pathways, the former of which is reminiscent of transposon silencing in higher eukaryotes. Nonetheless, the population of DNA elimination events dependent on PRC2 is complex, and we have only begun to scratch the surface of its involvement in this process. Further work is required to unravel the precise role of the complex in the removal of both new and ancient transposons.

### Limitations of the study

Due to technical limitations, further analysis into the effect of the PRC2 complex on nucleosomes was not possible beyond describing the density changes over IESs. Contamination from the maternal MAC makes interpretation of the nucleosome landscape for the rest of the genome impossible, because one cannot distinguish the reads originating from the two nuclei. Furthermore, the effect on IESs must be seen in light of the silencing efficiency of Pgm, because this determines how many reads contain IESs, irrespective of nucleosomes. Although our results suggest that PRC2-dependent heterochromatinization of IESs is unlikely, further experiments are required to precisely delineate the role of the PRC2 complex on chromatin.

## STAR★Methods

### Key resources table

**Table:** 

REAGENT or RESOURCE	SOURCE	IDENTIFIER
**Antibodies**		

Anti-H3K27me3	Millipore	Cat# 07-449; RRID: AB_310624
Anti-H3K9me3	Millipore	Cat# 07-442; RRID: AB_310620
Anti-H2AK119ub1	Cell Signaling Technology	Cat# 8240T RRID: AB_10891618
Anti-HA Affinity Matrix	Roche	Cat# 11815016001; RRID: AB_390914
goat anti-rabbit Alexa Fluor 546 Secondary antibody	Thermo Fisher Scientific	Cat# A-11071; RRID: AB_2534115
Rabbit Anti-HA-Tag Monoclonal Antibody	Cell Signaling Technology	Cat# 3724; RRID: AB_1549585
Mouse Anti-Myc-Tag Monoclonal Antibody	Cell Signaling Technology	Cat# 2276; RRID: AB_331783
Mouse anti-rabbit IgG-HRP	Santa Cruz Biotechnology	Cat# sc-2357; RRID: AB_628497
Goat anti-mouse IgG-HRP Polyclonal	Santa Cruz Biotechnology	Cat# sc-2005; RRID: AB_631736

**Bacterial and virus strains**		

*Escherichia coli* strain HT115 (DE3)	Gift from Eric Meyer (ENS, Paris)	HT115
Endura competent *E. Coli*	Lucigen	Cat# 60242-0
*Klebsiella pneumoniae* non-virulent strain, food source for *Paramecium*	Gift from Eric Meyer (ENS, Paris)	N/A

**Chemicals, peptides, and recombinant proteins**		

Wheat grass powder	Pines International, Lawrence, KS	N/A
β-sitosterol	Calbiochem, Millipore	Cat# 567152
DTT	Sigma-Aldrich	Cat# 3483-12-3
Complete EDTA-free protease inhibitor cocktail tablet	Roche	Cat# C762Q78
30% acrylamid:bisacrylamid 19:1	BioRad	Cat# 161-0154
Q5 high fidelity DNA polymerase	NEB	Cat# M0491L

**Critical commercial assays**		

RadPrime DNA Labeling System	Invitrogen	Cat# 18428011
EZ Nucleosomal DNA Prep Kit	ZYMO Research	Cat# D5220
QIAGEN Plasmid Midi Kit	QIAGEN	Cat# 12143
TruSeq DNA Nano Kit	Illumina	Cat# 20015965
TruSeq small RNA kit	Illumina	Cat# RS-200-0012

**Deposited data**		

sRNA-seq	This paper	PRJNA768531 (NCBI)
mRNA-seq	This paper	PRJNA768531 (NCBI)
Nucleosome DNA-seq	This paper	PRJNA768531 (NCBI)
Developing MAC DNA-seq	This paper	PRJNA768531 (NCBI)
Mass spectrometry	This paper	PXD028503 (PRIDE)
ChIP-seq of H3K9me3 and H3K27me3	From Frapporti et al., 2019	ERS3000371 to ERS3000378 (European Nucleotide Archive)
Developing MAC DNA sequencing of *PtCAF1* KD, *Dcl2/3* KD, *Dcl5* KD, *Dcl2/3/5* KD and *Ezl1* KD	Swart et al., 2017; Lhuillier-Akakpo et al., 2014; Sandoval et al., 2014	European Nucleotide Archive: ERS1033674 (*PtCAF1* KD), ERS1033670 (*Dcl2/3/5* KD), ERA309409 (*Ezl1* KD) NCBI: SRX387766 (*Dcl2/3* KD, *Dcl5* KD)

**Experimental models: Organisms/strains**		

*Paramecium tetraurelia*, strain 51	Gift from Eric Meyer (ENS, Paris)	N/A

**Oligonucleotides**		

17s rRNA probe	See sequences in Table S4	N/A
Primers of IES and transposon retention PCR	See sequences in Table S4	N/A
Primers of Actin	See sequences in Table S4	N/A

**Recombinant DNA**		

Recodonized PtCAF1-GFP-pUC57	This paper; see Table S2	N/A
Suz12-GFP-pGEM T	This paper	N/A
Suz12-Myc-pGEM T	This paper	N/A
Eed-GFP-pGEM T	This paper	N/A
Eed-Myc-pGEM T	This paper	N/A
Rnf1-GFP-pGEM T	This paper	N/A
Rnf2-GFP-pGEM T	This paper	N/A
Ezl1-Ku80c promoter-pGEM T	This paper	N/A
Suz12-Ku80c promoter-pGEM T	This paper	N/A
Eed-Ku80c promoter-pGEM T	This paper	N/A
Rnf2-Ku80c promoter-pGEM T	This paper	N/A
Recodonized PtCAF1-GFP Ku80c promoter-pGEM T	This paper	N/A

**Software and algorithms**		

after_ParTIES	Swart et al., 2017	https://github.com/gh-ecs/After_ParTIES
Salmon	Patro et al., 2017	https://github.com/COMBINE-lab/Salmon
DESeq2	Love et al., 2014	http://www.bioconductor.org/packages/release/bioc/html/DESeq2.html
Bowtie2	Langmead and Salzberg, 2012	http://bowtie-bio.sourceforge.net/bowtie2/index.shtml
IES nucleosome profiling pipelines	This paper; Zenodo	https://doi.org/10.5281/zenodo.6949086

**Other**		

Ultrafree-MC Centrifugal Filter	Millipore	Cat# UFC30GV25
Slide-A-Lyzer™ G2 Dialysis Cassettes	Thermo Fisher Scientific	Cat# 87723
Amicon Ultra-2 Centrifugation Filter Unit	Millipore	Cat# UFC200324

### Resource availability

#### Lead contact

Further information and requests for reagents and resources should be directed to the lead contact, Mariusz Nowacki (mariusz.nowacki@unibe.ch).

#### Materials availability

This study did not generate any unique reagents.

### Experimental model and subject details

*Paramecium tetraurelia* strain 51, mating type seven, was used to perform the experiments. Cells were cultured at 27°C in wheat grass powder (WGP) medium (Pines international, Lawrence, KS) bacterized with *Klebsiella pneumoniae* and supplemented with 0.8 mg/mL β-sitosterol (Merck) as previously described (Beisson et al., 2010b). Autogamy was induced by starvation.

### Method details

#### Constructs

The regions of *PtCAF1* (31–952 nt), *Suz12* (1–700 nt), *Eed* (1–900 nt), *Rnf1* (1–1,000 nt) and *Rnf2* (1–1,677 nt) were cloned between the two inverted T7 promoters of L4440 vector to make the silence constructs. The constructs of *Ezl1*, *Ptiwi01*, *Ptiwi09*, *Dcl2*, *Dcl3* and *Pgm* are same with the published papers (Lhuillier-Akakpo et al., 2014; Bouhouche et al., 2011; Baudry et al., 2009; Lepere et al., 2009). Empty vector of L4440 was used as the negative control.

The full gene and both flanking regions of *Suz12* (311 bp upstream and 293 bp downstream), *Eed* (338 bp upstream and 288 bp downstream), *Rnf1* (450 bp upstream and 327 bp downstream), and *Rnf2* (366 bp upstream and 298 bp downstream) were cloned into the pGEM-T vector. For *Suz12*, *Eed* and *Rnf2*, the codon optimized GFP, FlagHA or Myc fragments were inserted immediately after the initiating ATG. For *Rnf1*, the GFP was inserted before the terminating TGA. The constructs of *PtCAF1*, *Ezl1* and *Ptiwi09* are the same with the published papers (Ignarski et al., 2014; Lhuillier-Akakpo et al., 2014; Bouhouche et al., 2011). To make the RNAi-resistant *PtCAF1*, the silencing region of *PtCAF1* was recodonized without changing the amino acid sequence (see the sequence in Table S2). To delay the expression of *Ezl1*, *PtCAF1*, *Suz12*, *Eed* and *Rnf2,* their flanking regions were replaced by the flanking sequences of *Ku80c* (338 bp upstream and 381 bp downstream).

#### Macronuclear transformation by microinjection

Constructs were linearized in the backbone of the vector, purified with phenol chloroform (pH 8) and Ultrafree-MC Centrifugal Filter (Millipore), and injected to the macronucleus of 4–10 division old vegetative cells as described in Beisson et al. (2010a). Successful injection was confirmed by dot blot.

#### Dot blot

Dot blot was performed as described in Arambasic et al. (2014). Briefly, 400 μL cells at a concentration of ∼1,000 cells/mL were lysed at 68°C for 30 min with the addition of 50 μL 0.5 M EDTA and 50 μL 4 M NaOH. The DNA was then transferred to a charged nylon membrane (Amersham Hybond-XL, GE Healthcare Life Sciences) and hybridized with FlagHA or GFP probes labeled with α-P^32^ dATP using the RadPrime DNA Labeling System (Invitrogen). The membrane was hybridized in Church buffer (1% BSA, 0.5 M NaPO_4_ (pH 7.2), 7% SDS, 1 mM EDTA) containing the probe, and incubated overnight at 60°C. Membranes were exposed on a phosphor screen (Amersham) and visualized on a Typhoon FLA 7000 (GE Healthcare).

#### Gene silencing

*Escherichia coli* strain HT115 (DE3) was used to produce double-stranded RNA (dsRNA) against the target genes and silencing by feeding was performed as previously described (Beisson et al., 2010c). Each silencing was performed at least three times. Briefly, bacteria were cultured in LB overnight, then diluted in 1x WGP medium (1:100) and incubated overnight. The following day, they were diluted with 1x WGP medium to the final volume with an OD_600_ of 0.04 and allowed to reach an OD_600_ between 0.07 and 0.1, before IPTG (0.4 mM) was added to induce the production of dsRNA. After at least 4 h, β-sitosterol (0.8 μg/mL) was added, the cultures cooled down to 27°C and Paramecia seeded into the silencing medium at a concentration of ∼200 cells/mL.

#### Genomic DNA extraction

Total genomic DNA for PCR was extracted from 100 mL of post-autogamous cells using GenElute Mammalian Genomic DNA Miniprep Kit (Sigma-Aldrich) or DNeasy Blood & Tissue Kit (Qiagen) following the manuals.

#### Macronuclear DNA extraction

DNA for high-throughput sequencing was prepared as in (Arnaiz et al., 2012). Two to three million post-autogamous cells were collected and washed twice in 10 mM Tris (pH 7.4). All the following steps were performed at 4°C unless otherwise noted. The cell pellets were lysed with a Potter-Elvehjem homogenizer in 2.5 volumes of lysis buffer 1 (0.25 M sucrose, 10 mM MgCl_2_, 10 mM Tris (pH 6.8), 0.2% Nonidet P-40). After two washes with wash buffer (0.25 M sucrose, 10 mM MgCl_2_, 10 mM Tris (pH 7.4)), the pellet was resuspended in three volumes of sucrose buffer (2.1 M sucrose, 10 mM MgCl_2_, 10 mM Tris (pH 7.4)). A sucrose gradient was prepared in a centrifuge tube (344060, Beckman Coulter) by carefully layering the samples on top of 3 mL of sucrose buffer, followed by filling up the tube with wash buffer. Ultracentrifugation was then ran at 35,000 rpm for 1 h and 4°C, using a Beckman Optima L-90K Ultracentrifuge. After ultracentrifugation, the pellet containing the nuclei was resuspended in 500 ul of 10 mM Tris (pH 7.4) with 10 mM MgCl_2_. To lyse the nuclei, 3 volumes of Lysis buffer 2 (0.5 M EDTA (pH 9), 1% N-lauryl sarcosine sodium, 1% SDS, 1 mg/mL proteinase K) was added and the solution incubated overnight at 55°C. After lysis, the DNA was extracted with phenol chloroform, and dialysis performed with a Slide-A-Lyser Dialysis cassette (Thermo Scientific) in 10 mM Tris (pH 8) with 1 mM EDTA (pH 8), exchanged three times (Intervals: 2h, 2h, overnight). Finally, the DNA was concentrated with a Amicon Ultra-2 Centrifugation Filter Unit (Millipore). The Illumina TruSeq DNA PCR-Free kit was used for library preparation and paired-end 2 × 150 bp sequencing performed on a NovaSeq. Library preparation and whole-genome sequencing was performed at the Next Generation Sequencing (NGS) Platform of the University of Bern, Switzerland.

#### Nucleosomal DNA extraction

Around 1.5 million cells were harvested and the nuclei isolated as described for macronuclear DNA isolation. After ultracentrifugation, the pellet was resuspended in 1x PBS and washed twice with 1 mL cold 1x PBS before proceeding with the nucleosomal DNA extraction. The following steps were optimized from the standard protocol of the EZ Nucleosomal DNA Prep Kit (ZYMO Research), using the Atlantis dsDNase (ZYMO Research). Nuclei were incubated with 1 mL chilled Nuclei Prep Buffer on ice for 5 min and then washed twice with 1 mL Atlantis Digestion Buffer. After centrifugation, the supernatant was discarded, and the nuclei were gently resuspended in 200 μL Atlantis Digestion Buffer with the addition of 17 μL Atlantis dsDNase. To digest the DNA, the nuclei were incubated at 42°C for 25 min and the reaction stopped by the addition of 1x MN Stop Buffer. Nucleosomal DNA was extracted using the column supplied in the kit following the manuals. From the same sample at the same time, macronuclear DNA isolation was performed and sequenced as described before, as a non-digested control. Library preparation and sequencing was performed at Fasteris (Geneva, Switzerland), according to the illumina Truseq Nano kit (without fragmentation or size selection), and paired-end 2 × 150 bp sequencing done on a Novaseq.

#### IES and transposon retention PCR

GoTaq DNA polymerase (Promega) or Taq plus master mix (Vazyme) were used for IES and transposon retention PCR with primers available in the supplementary file (Table S4).

#### Cytological staging

The developmental timepoints were assessed by 4,6-diamidino-2-phenylindole (DAPI)-staining and microscopy, staging a minimum of 100 cells. In this study we collected Early (in which approximately 30–40% of cells have a fragmented maternal MAC, the rest vegetative), and Late (in which nearly all cells are fragmented and have visible new MACs).

#### Immunofluorescence

Cells were permeabilized with 1% Triton X-100 in 1x PHEM buffer (10 mM EGTA, 25 mM HEPES, 2 mM MgCl_2_, 60 mM PIPES (pH 6.9)) for 10 min and then fixed with 2% Paraformaldehyde for 10 min. The cells were blocked in 3% BSA in TBSTEM buffer (10 mM EGTA, 2 mM MgCl_2_, 0.15 M NaCl, 10 mM Tris, 1% Tween 20 (pH 7.4)) for an hour. After blocking, Anti-trimethyl-Histone H3 (Lys27) (07-449, Millipore), anti-trimethyl-Histone H3 (Lys9) (07-442, Millipore) or anti-ubiquityl-Histone H2A (Lys119) (8240T, Cell Signaling Technology) was used as the primary antibody at a dilution of 1:200 and incubated overnight at 4°C. After washing twice with 3% BSA in TBSTEM, the cells were incubated for an hour with goat anti-rabbit Alexa Fluor 546 Secondary antibody (A-11071, Invitrogen) at a dilution of 1:4,000. The cells were washed twice with 1x PBS, spread on glass slides and mounted with ProLong Glass Antifade Mountant (P36980, Invitrogen), formulated with the blue DNA stain NucBlue.

#### GFP localization and imaging

Cells were collected, washed twice in 10 mM Tris (pH 7.4) and fixed in 70% EtOH. For imaging, cells were washed thrice with 1x PBS, before the staining with DAPI. Imaging was performed with a Axiovert A1(Zeiss) or Axio Imager.D2 (Zeiss) and processed with the ZEN 2 software (Zeiss).

#### Survival test

Thirty post-autogamous cells were transferred to individual wells with bacterized 0.2x WGP supplemented with 0.8 mg/mL of β-sitosterol (Merck) and counted for three or four consecutive days to evaluate the survival of the progeny. By comparing with the control, cells were divided into healthy, sick and dead categories.

#### RNA extraction and sequencing

600,000 cells were collected, washed twice with 10 mM Tris (pH 7.4) and the cell pellets frozen in liquid nitrogen. Total RNA was extracted with TRIzol (Sigma-Aldrich) following the TRIzol reagent BD protocol.

For RNAseq: library preparation using illumina TruSeq stranded RNA kit and paired-end 2 × 150 bp sequencing on a NovaSeq were performed at the Next Generation Sequencing (NGS) Platform at the University of Bern, Switzerland.

For small RNA seq: small RNAs were size selected by polyacrylamide gel selection (17–35 nt) and library preparation performed using the illumina TruSeq small RNA kit. Single-end 1 × 75 bp sequencing was performed on a NextSeq. Size selection, library preparation and sequencing were performed at Fasteris (Geneva, Switzerland).

#### Northern blot

Total RNA (∼10 μg) was separated in a 1.2% denaturing agarose gel with 2.2 M formaldehyde and transferred to a charged nylon membrane (Amersham Hybond-XL, GE Healthcare Life Sciences) by capillary blotting in 20x saline-sodium citrate buffer. The membrane was UV crosslinked twice at 120,000 μJ/cm^2^. A probe specific for *PtCAF1* (1,143 to 1,298 nt) was labeled with α-P^32^ dATP using RadPrime DNA Labeling System (Invitrogen). An rRNA probe was used as the loading control (sequence shown in Table S4). Hybridization and visualization were performed as for Dot blot.

#### Immunoprecipitation (IP) and Western blot

Around 1.2 million cells were collected, washed twice with 10 mM Tris (pH 7.4), followed by two washes with 1x PBS and the supernatant removed. The pellet was resuspended in 2 mL lysis buffer (50 mM Tris (pH 8.0), 150 mM NaCl, 5 mM MgCl_2_, 1 mM DTT, 1x complete EDTA-free protease inhibitor cocktail tablet (Roche), 1% Triton X-100, 10% glycerol) and sonicated at 55% amplitude for 15 s using a Branson digital sonifier. The soluble fraction was separated by centrifugation and 1 mL was incubated overnight at 4°C with 50 μL Anti-HA Affinity Matrix (Roche) which had been washed thrice with IP buffer (10 mM Tris (pH 8.0), 150 mM NaCl, 1 mM MgCl_2_, 0.01% NP40, 5% glycerol). After overnight incubation, the beads were washed six times with IP buffer and the supernatant removed. The beads fraction was used for further experiments.

For Western blot, the samples were separated by SDS-PAGE electrophoresis and transferred to a nitrocellulose membrane (Amersham Protran, GE Healthcare Life Sciences) by wet transfer. The membrane was blocked in blocking buffer (10% skim milk in 1x PBS with 0.1% Tween 20) for 1 h at room temperature and incubated overnight at 4°C with the primary antibody solution. Between the primary and secondary antibody incubations, three washes with PBST for 5 min each were performed. Secondary antibody incubation was performed for 1 h at room temperature, followed by three washes with PBST and one wash with PBS. The HRP substrate (Immobilon Forte, Millipore) was applied to the membrane and it was scanned on an Amersham 600 (GE Healthcare Life Sciences). Primary antibodies used were rabbit anti-HA (3724S, Cell Signaling Technology) at a dilution of 1:1,000, or mouse anti-Myc antibody (2276S, Cell Signaling Technology) at a dilution of 1:1,000. Secondary antibodies were mouse anti-Rabbit IgG-HRP (sc-2357, Santa Cruz) and goat anti-Mouse IgG-HRP (sc-2005, Santa Cruz), at a dilution of 1:10,000. All antibodies were diluted in blocking buffer.

### Quantification and statistical analysis

#### Mass spectrometry analysis

For mass spectrometry, proteins were separated by SDS-PAGE electrophoresis and stained with InstantBlue (Expedeon), before the entire sample was cut into cubes and stored in 20% Ethanol until processing. The mass spectrometry analysis was performed at the Proteomics Mass Spectrometry Core Facility (PMSCF) of the University of Bern, Switzerland. The gel pieces were reduced, alkylated and digested by trypsin. The digests were analyzed by liquid chromatography (LC)-MS/MS (PROXEON coupled to a QExactive HF mass spectrometer, Thermo Fisher Scientific) with one injection of 5 μL digests. Peptides were trapped on a μPrecolumn C18 PepMap100 (5 μm, 100 Å, 300 μm × 5 mm, ThermoFisher Scientific, Reinach, Switzerland) and separated by backflush on a C18 column (5 μm, 100 Å, 75 μm × 15 cm, C18) by applying a 40-min gradient of 5% acetonitrile to 40% in water, 0.1% formic acid, at a flow rate of 350 nL/min. The Full Scan method was set with resolution at 60,000 with an automatic gain control (AGC) target of 1E06 and maximum ion injection time of 50 ms. The data-dependent method for precursor ion fragmentation was applied with the following settings: resolution 15,000, AGC of 1E05, maximum ion time of 110 milliseconds, mass window 1.6 m/z, collision energy 27, under fill ratio 1%, charge exclusion of unassigned and 1+ ions, and peptide match preferred, respectively.

The mass spectrometry data was interpreted with MaxQuant version 1.5.4.1 using a concatenated target and reverted decoy protein sequence database of *Paramecium tetraurelia* (v1.99.27 downloaded from the Centre National de Séquençage website, https://www.cea.fr/) enriched with some common contaminating proteins applying full trypsin specificity, allowing for up to 3 missed cleavages, variable modification on protein N-termini with acetylation, oxidation on methionine, deamidation on asparagine and glutamine, and static modification with carbamidomethylation on cysteine, with a mass accuracy of 10 ppm for precursor and 20 ppm for fragment ions. Only proteins with at least 2 peptides identified with a 1% FDR level were accepted. The match between run option was activated but interpretation between different sample sets hindered by non-consecutive fraction numbering. Differential protein abundance analysis was based on the mean of the top3 (or LFQ) peptide intensities. For this, peptide intensities were normalized using the variance stabilization transformation (bioconductor package vsn). If no protein was found in a sample, the reported intensity was set to zero. Differential analysis using the empirical Bayes statistics from the bioconductor limma package was performed with the false discovery rate set at 0.01.

#### Protein domain prediction

The protein domain was predicted by InterPro (https://www.ebi.ac.uk/interpro/), NCBI Conserved Domain Research (https://www.ncbi.nlm.nih.gov/Structure/cdd/wrpsb.cgi), HHpred (https://toolkit.tuebingen.mpg.de/tools/hhpred) and Phyre2 (http://www.sbg.bio.ic.ac.uk/phyre2/html/page.cgi?id=index) (Blum et al., 2021; Lu et al., 2020; Zimmermann et al., 2018; Kelley et al., 2015).

#### Reference genomes

The following reference genomes were used in the IES analyses and were used for read mapping:

MAC: http://paramecium.cgm.cnrs-gif.fr/download/fasta/ptetraurelia_mac_51.fa.

MAC+IES: http://paramecium.cgm.cnrs-gif.fr/download/fasta/ptetraurelia_mac_51_with_ies.fa.

TE: https://paramecium.i2bc.paris-saclay.fr/files//Paramecium/tetraurelia/51/annotations/ptetraurelia_mic2/ptetraurelia_TE_consensus_v1.0.fa.

OES: https://paramecium.i2bc.paris-saclay.fr/download/Paramecium/tetraurelia/51/sequences/pteraurelia_mic2.fa.

IES retention scores and correlation plots.

ParTIES was used to calculate the IES retention scores (IRSs) for *Rnf1*, *Rnf2*, *Eed*, and *Suz12*. In brief, ParTIES counts mapped reads with unexcised IESs (IES+) and excised IESs (IES-) to calculate an IES’s retention score, IRS = IES+ ÷ (IES+ + IES-).

Correlation plots of the impact of experimental silencings on IES retention were performed using After_ParTIES (Swart et al., 2017).

#### Small RNA-seq mapping

Single-end sRNA reads were separated (∼15–45 nt) and then mapped with Bowtie2 (v2.3.3; (Langmead and Salzberg, 2012)) using the default parameters. These size-selected reads were mapped to the following reference data sets: the somatic MAC genome (*P. tetraurelia* strain 51); IESs from the MAC+IES genome; annotated reference TEs; the germline MIC genome (referred to as OESs); the L4440 vector sequence (Addgene). Mapped sRNA reads were then normalized by the total number of reads to generate histograms based on their putative sources.

#### RNA-seq data analysis

Paired end reads were mapped against the *P. tetraurelia* transcriptome and annotated TEs with Salmon (v2.5.1; (Patro et al., 2017)) with the “--validateMappings --seqBias --gcBias --posBias” flags, with the somatic and germline genomes used as decoys. The abundance estimates were used as the input for DESeq2 (Love et al., 2014). Genes and transposons with fewer than 20 normalized counts in any given timepoint/sample were filtered out prior to analysis. Differentially expressed genes/TEs in the PtCAF1-KDs were identified as those with an adjusted p value less than 0.01 and with at least a two-fold change relative to the corresponding control time point. Genes were classified as upregulated (fold change ≥2) or downregulated (fold change ≤½).

#### Nucleosome density analysis

HISAT2 (Kim et al., 2019) was used for nucleosomal and MAC DNA read mapping with parameter “--min-intronlen 24” and “--max-intronlen 20000”. For nucleosome profiling “properly paired” (as defined by the samtools flag “2”) paired-end reads with an outer distance between 125 and 175 bp, in the range expected for mononucleosomes were selected for further analysis. Bedtools (Quinlan and Hall, 2010) was used to extract reads overlapping IESs by at least 9 bp with the parameters “-f 0.06 -split”. htseq-count from the HTSeq package (Anders et al., 2015) was used to count IES-matching reads. The combined output and shell script for this procedure are provided in the supplement (Data S1) and GitHub (https://github.com/Swart-lab/IES_nuc_density).

Nucleosome profiling reads across IESs were normalized to total DNA according to the following (subscript e = empty vector control; subscript p = PtCAF1-KD):

D_e_ = number of mapped DNA reads from the empty vector control knockdown.

N_e_ = number of mapped nucleosomal reads from the empty vector control knockdown.

D_p_ = number of mapped DNA reads from the PtCAF1 knockdown.

N_p_ = number of mapped downsampled nucleosomal reads from the PtCAF1 knockdown

r_e_ = (n_e_/N_e_) ÷ (d_e_/D_e_).

r_p_ = (n_p_/N_p_) ÷ (d_p_/D_p_).

We refer to these fractions as “DNA-seq normalized nucleosome densities”. These are dimensionless quantities as implicit DNA-seq and nucleosome profiling IES length normalizations of densities (reads per bp) cancel each other out by division.

Total mapped reads of the DNA-seq libraries were very similar (19274110 and 19338954 for control vs. PtCAF1-KD), so no normalization for differences in library sizes/mapped reads were made. Total mapped nucleosomal reads differed more (10195480 vs. 11930456), so we randomly downsampled the reads mapped to IESs from the PtCAF1-KD (providing the switch -s 0.8545, with suitable size factor to samtools) to obtain counts.

KS statistics with associated p values for comparing the distributions of DNA-seq normalized nucleosome densities were calculated using the ks_2samp function from SciPy’s statistics library (https://docs.scipy.org/doc/scipy/reference/generated/scipy.stats.ks_2samp.html).

## Data Availability

• All the DNA and RNA sequencing data have been uploaded to NCBI under the bioproject PRJNA768531. The mass spectrometry results were submitted to PRIDE with project accession PXD028503. Accession numbers are listed in the key resources table. This paper analyzes existing, publicly available data. These accession numbers for the datasets are listed in the key resources table. • All original code has been deposited at Zenodo and is publicly as of the date of publication. DOIs are listed in the key resources table. • Any additional information required to reanalyze the data reported in this paper is available from the lead contact upon request.

## Supplemental information

Document S1. Figures S1–S7 and Tables S1, S2, and S4

Table S3. Differentially expressed genes on PtCAF1-KD, related to Figure 3

Data S1. Output of nucleosome DNA analysis, related to STAR Methods

Document S2. Article plus supplemental information
