# Supplementary material for: A small RNA-guided PRC2 complex eliminates DNA as an extreme form of transposon silencing
Source: Cell Rep. 2022 Aug 23;40(8):111263. doi: 10.1016/j.celrep.2022.111263 (PMC10073204; doi:10.1016/j.celrep.2022.111263)
Supplement: Document S1. Figures S1–S7 and Tables S1, S2, and S4 [file mmc1.pdf]

Cell Reports, Volume *40*

Supplemental information

A small RNA-guided PRC2 complex eliminates DNA as an extreme form of transposon silencing

Chundi Wang, Therese Solberg, Xyrus X. Maurer-Alcalá, Estienne C. Swart, Feng Gao, and Mariusz Nowacki

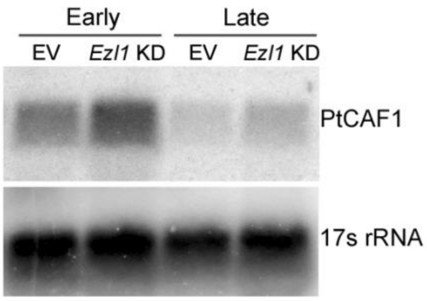

Figure S1: Ezl1 is not required for the transcription of *PtCAF1*. Related to Figure 1.
Northern blot using a probe against *PtCAF1* in early and late timepoints of empty vector (EV) and *Ezl1* silencing. *17s rRNA* was used as a loading control.

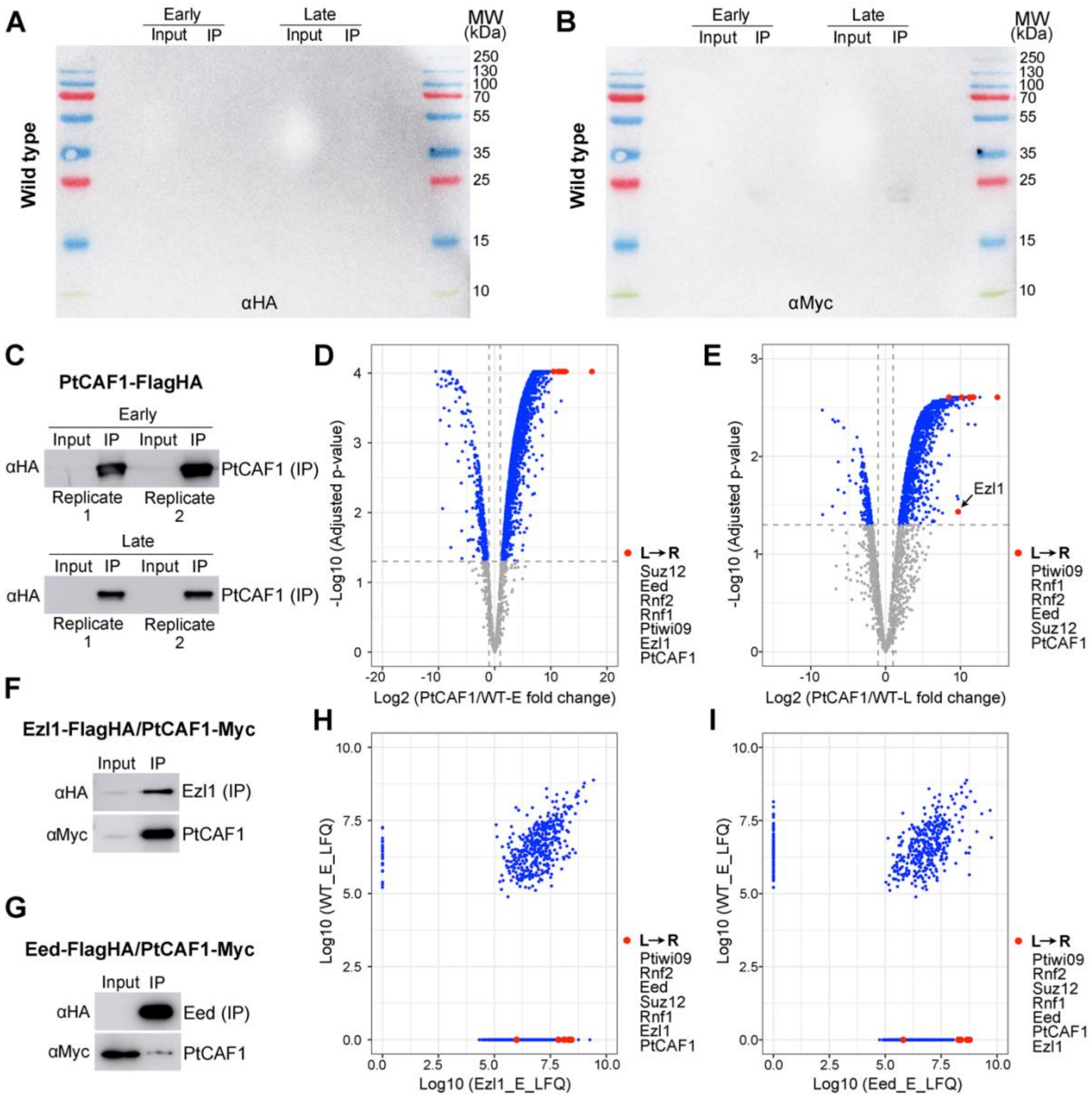

Figure S2: Western blot and mass spectrometry results PtCAF1, Ezl1 and Eed immunoprecipitated proteins. Related to Figure 1.

(A-B) Western blot of HA-immunoprecipitated proteins in wild type incubated with anti-HA or anti-Myc antibodies. αHA: incubated with anti-HA antibody. αMyc: incubated with anti-Myc antibody. (C, F-G) Western blot of input and HA-immunoprecipitated proteins using anti-HA or anti-Myc antibodies to detect PtCAF1, Ezl1 or Eed. (D-E) Volcano plots to show the mass spectrometry results of PtCAF1 (D: early timepoint; E: late timepoint) co-immunoprecipitated proteins. (H-I) Scatter plots to show the mass spectrometry results of Ezl1 (early timepoint) and Eed (early timepoint) co-immunoprecipitated proteins, with wild type as the negative control. X and Y axis are the log10 of LFQ values. Red dots represent Ezl1, Suz12, Eed. PtCAF1, Rnf1, Rnf2 and Ptiwi09 with their order at bottom right corner of each subplots (from left to right).

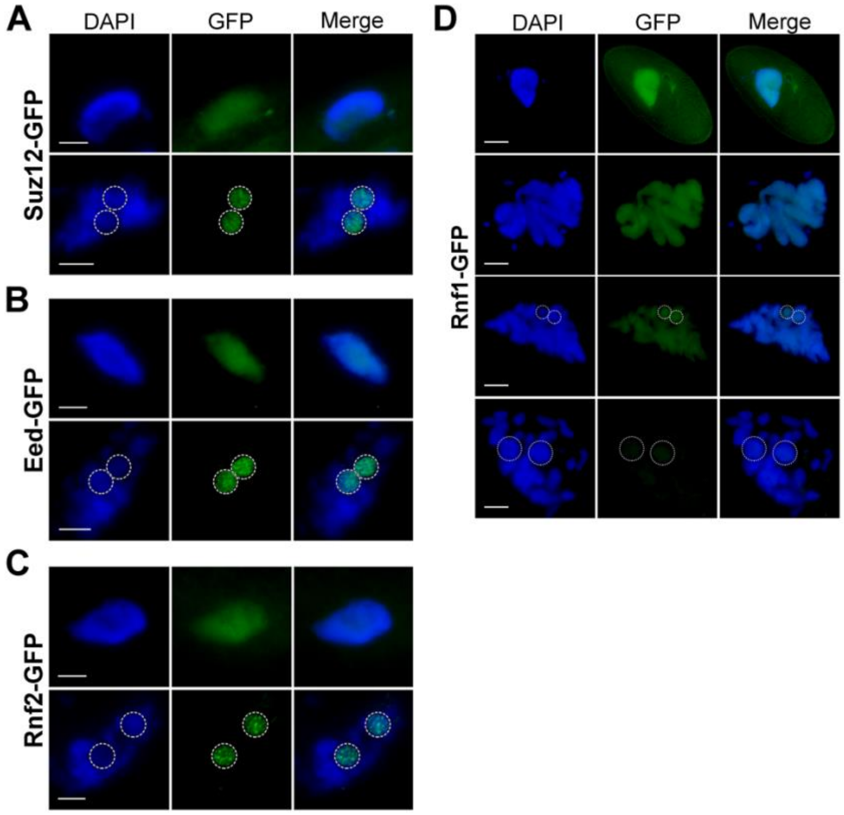

Figure S3: Localization of Suz12, Eed, Rnf1 and Rnf2. Related to Figure 1.
New MACs are indicated with white dashed circles. Scale bar: 10µm.

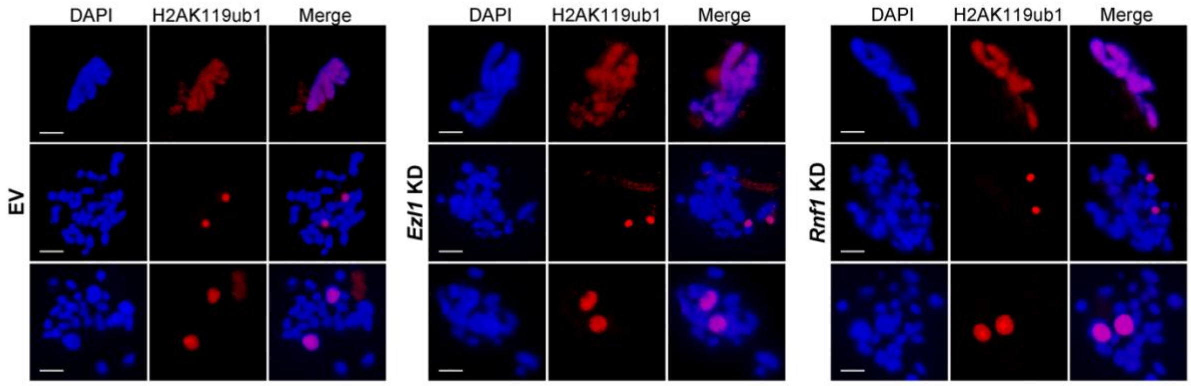

Figure S4: Distribution of H2AK119ub1 in control, *Ezl1* KD and *Rnf1* KD cells during sexual development. Related to Figure 1.

EV: empty vector silencing, control. Scale bar: 10μm.

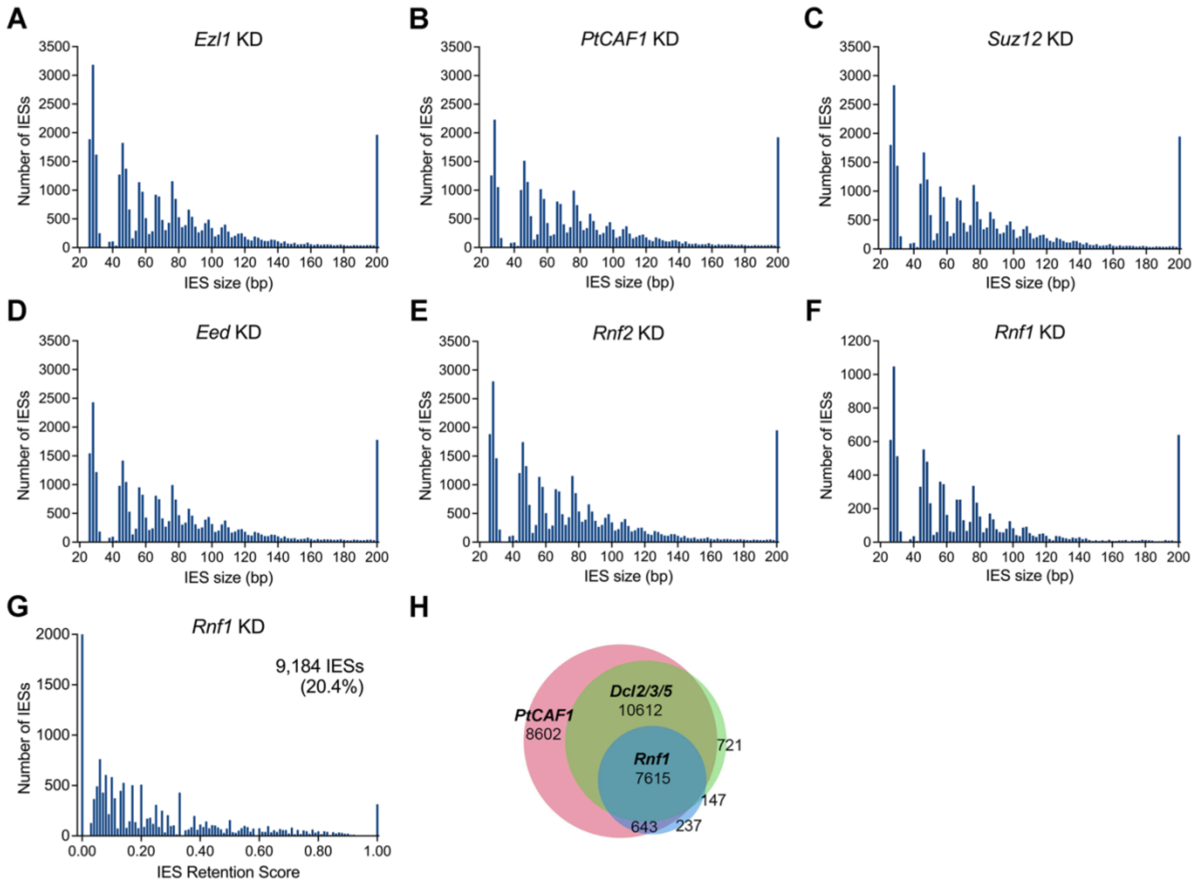

Figure S5: IES size distribution after depletion of PRC2 subunits. Related to Figure 2.
(A-F) Size distribution of the retained IESs (Retention score >= 0.1) after *Ezl1*, *PtCAF1*, *Suz12*, *Eed*, *Rnf2* and *Rnf1 KD*. (G) IES retention score after *Rnf1* depletion. The numbers under the gene name show the number and percentage of IESs whose retention score are equal or greater than 0.1. (H) Venn diagram depicting shared IESs retained after *PtCAF1*, *Dcl2/3/5* and *Rnf1* depletion.

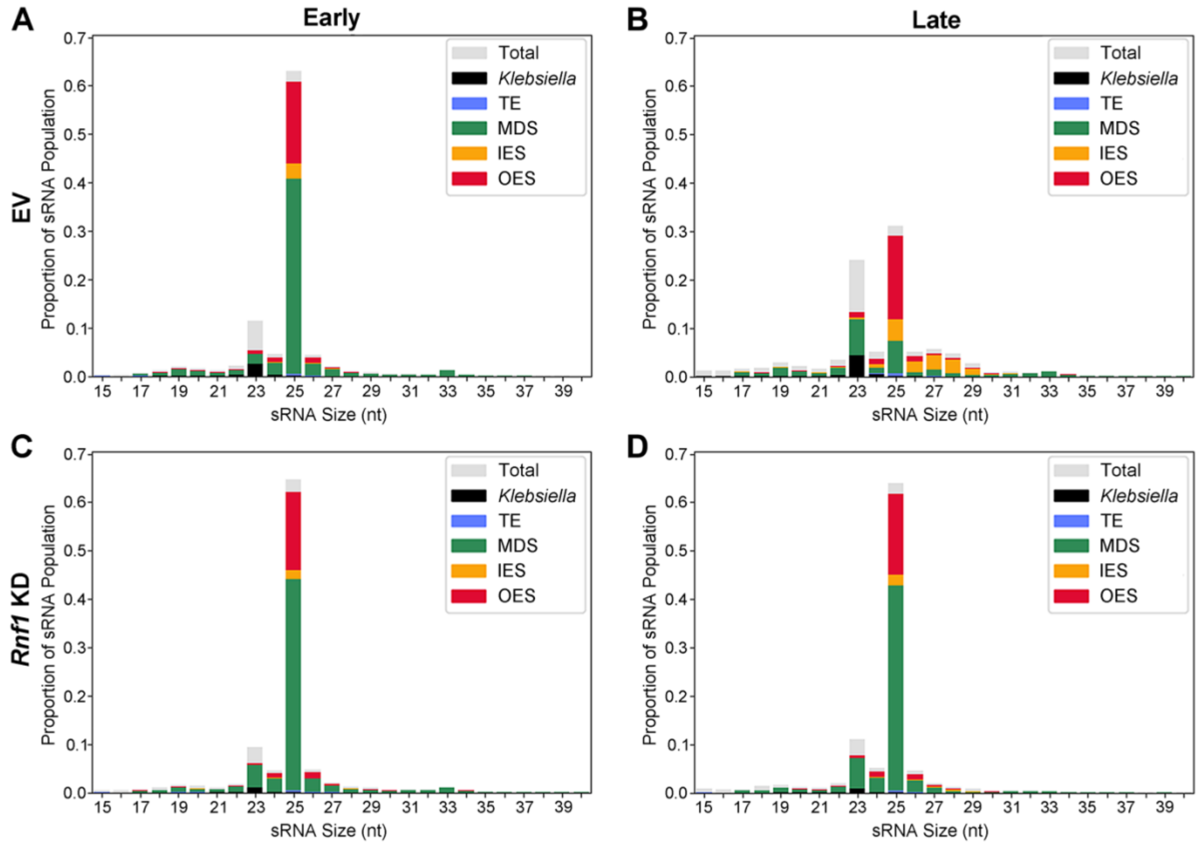

Figure S6: Small RNA mapping in control and *Rnf1*-KD in early and late timepoints. Related to Figure 3.

EV: empty vector silencing (control); TE: transposable element; MDS: macronuclear destined sequence (MAC-matching); IES: internal eliminated sequence; OES: other eliminated sequence.

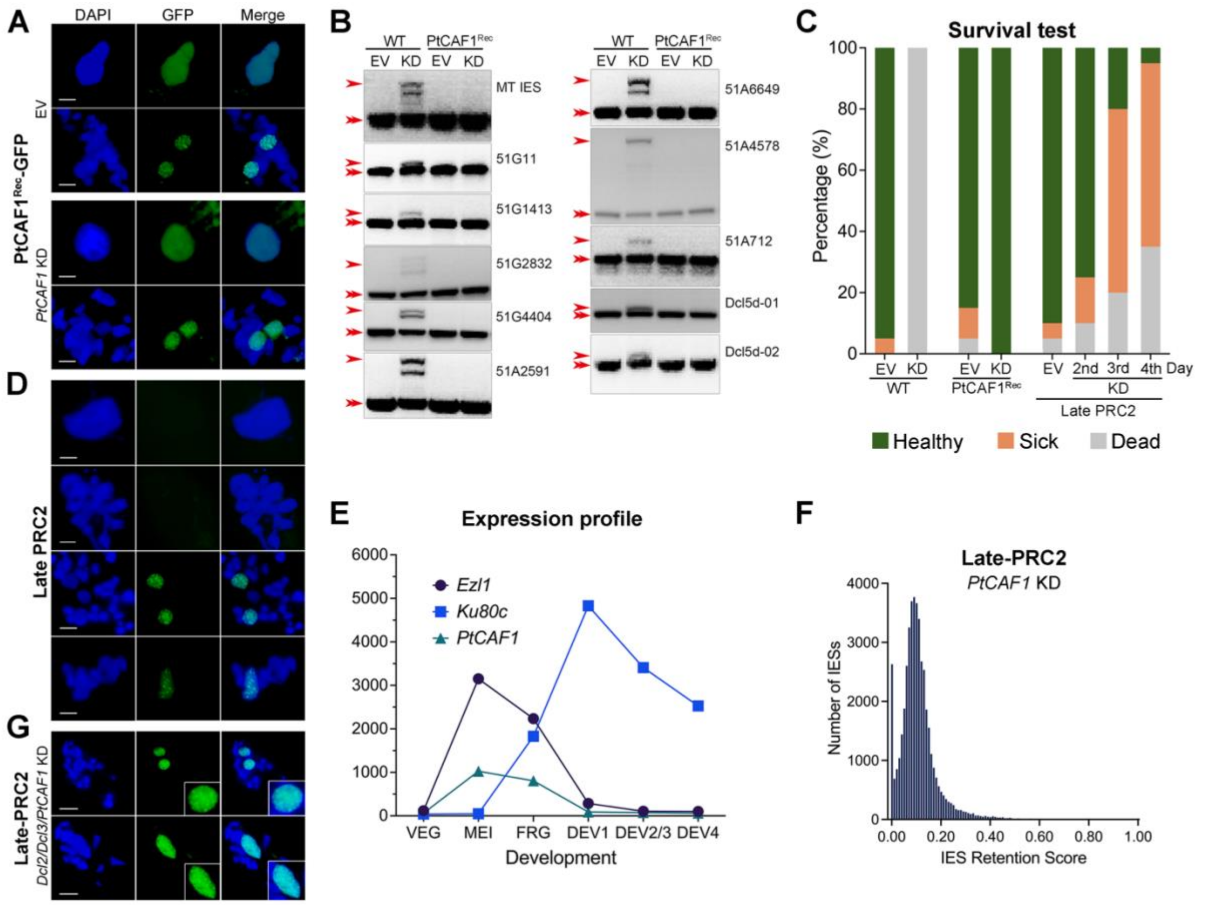

Figure S7: Procedures to get the new MAC specific PRC2. Related to Figure 4.
(A) Localization of recodonized PtCAF1-GFP in control (empty vector silencing, EV) and endogenous *PtCAF1* KD. (B) IES retention PCRs of control (EV) and *PtCAF1* KD, in wild type and cells expressing the recodonized PtCAF1-GFP. Single arrowheads indicate the bands with IES and the double arrowheads indicate the bands without IES. (C) Survival test of wild type, recodonized PtCAF1 and late PRC2 injected cells in EV and *PtCAF1* KD. For wild type and recodonized PtCAF1 injected cells, three days after completion of development were monitored. For late PRC2 injected cells, four days were monitored continuously, in which control cells have similar results during these four days with only the last day result shown. Green: healthy; Pink: sick; Gray: dead. EV: empty vector silencing; KD: *PtCAF1* KD; WT: wild type; PtCAF1^{Rec}: cells injected with recodonized PtCAF1-GFP; Late PRC2: cells injected with the mix of recodonized PtCAF1-GFP, Ezl1, Suz12, Eed and Rnf2 (all are regulated by *Ku80c* promoter). (D) Localization of recodonized PtCAF1-GFP regulated by *Ku80c* promoter in late PRC2 injected cells. (E) Expression profile of *Ku80c*, *Ezl1 and PtCAF1* genes. (F) IES retention scores of *PtCAF1* KD in late PRC2 injected cells. (G) Localization of recodonized PtCAF1-GFP regulated by *Ku80c* promoter in late PRC2 injected cells at late (new MACs) stage after *Dcl2/Dcl3/PtCAF1* KD. Scale bar: 10μm.

Table S1. Top hits in the mass spectrometry of immunoprecipitated PtCAF1, Ezl1 and Eed. Related to Figure 1.

	ID. (ParameciumDB)	Molecular Weight (kDa)	Unique peptides	PtCAF1 (LFQ)				Ezl1 (LFQ)	Eed (LFQ)	Wild type (LFQ)			
				Early		Late		Early	Early	Early		Late	
				Rep.1	Rep.2	Rep.1	Rep.2			Rep.1	Rep.2	Rep.1	Rep.2
PtCAF1	PTET.51.1.P0780031	45.5	51	$6.1*10^9$	$6.5*10^9$	$1.5*10^9$	$1.7*10^9$	$2.8*10^8$	$4.6*10^8$	-	-	-	-
Ezl1	PTET.51.1.P1740049	69.6	41	$1.6*10^8$	$2.2*10^8$	$4.0*10^8$	$6.5*10^8$	$2.4*10^8$	$6.0*10^8$	-	-	-	$1.1*10^7$
Suz12	PTET.51.1.P0190277	34.5	20	$8.1*10^7$	$6.2*10^7$	$1.4*10^8$	$1.8*10^8$	$1.3*10^8$	$1.9*10^8$	-	-	-	-
Eed	PTET.51.1.P0240079	43.8	27	$5.9*10^7$	$9.4*10^7$	$1.3*10^8$	$1.6*10^8$	$1.3*10^8$	$2.4*10^8$	-	-	-	-
Rnf1	PTET.51.1.P0570234	62.5	49	$1.4*10^8$	$1.0*10^8$	$9.8*10^7$	$8.4*10^7$	$2.1*10^8$	$2.1*10^8$	-	-	-	-
Rnf2	PTET.51.1.P1190062	74.6	61	$8.9*10^7$	$7.8*10^7$	$1.6*10^8$	$1.2*10^8$	$7.3*10^7$	$1.9*10^8$	-	-	-	-
Ptiwi09	PTET.51.1.P0660118	87.5	60	$3.0*10^8$	$3.8*10^8$	$2.2*10^7$	$2.5*10^7$	$3.8*10^7$	$2.1*10^7$	-	-	-	-

ID: identity number; LFQ: label-free quantitation; Rep.: replicate; “-“: not detected.

Table S2. Sequence of recodonized *PtCAF1* regulated by *Ku80c* promoter with GFP N-terminally tagged. Related to STAR methods.

Recodonized *PtCAF1* with *Ku80c* Promoter and GFP tag
CAAAAAACTCCATTAGTTGTTAACATGCATTTCTGATTGGTTGAGGGACTCTGTCTTTATTGATTT<br>CTTTAACACAACTTTCAACTTCTTTATAGTTATTTAAGCCGCGGTCCTCAGCACAATCAGAACAAA<br>TGAGTTTATAGCCAAGGGGGACATTTGGGGAATAACTCATGTATCTAATGGGCTTGTTGTGATAT<br>GGACATAGTAATTTCTTGATGTAATCGATGATAAGTTAATTTTCTTAGATTTACATTAAATAGATAAA<br>TAATACAGGCAATTTTTTAATATACAAATTTCAATAATGAATTTGTAGATATATATAAAAAATAAGTGC<br>AAAGATGAATTCTAGAGGAGAAGAACTTTTCACTGGTGTTGTTCCAATTCTTGTTGAACTTGATG<br>GTGATGTTAATGGACATAAATTTTCTGTCTCTGGTGAGGGTGAAGGTGATGCAACTTATGGAAAA<br>TTAACCTTAAAATTTATTTGCACTACTGGAAAATTACCTGTTCCATGGCCAACACTTGTCACTACT<br>TTAACTTATGGAGTCCAATGTTTTTCAAGGTACCCTGACCACATGAAACAACATGACTTTTTCAA<br>ATCTGCCATGCCAGAAGGATATGTCCAAGAAAGAACTATATTCTTCAAAGATGATGGAAACTACA<br>AGACAAGAGCTGAAGTCAAATTTGAAGGAGATACCCTTGTCAATAGAATTGAGCTTAAAGGAATT<br>GATTTTAAAGAAGATGGAAACATTTTAGGCCATAAATTGGAATACAACTATAACTCACATAATGTAT<br>ACATCATGGCAGACAAACAAAAAAATGGAATCAAAGTCAACTTCAAAATTAGACACAACATTGAA<br>GATGGATCAGTTCAATTAGCAGACCATTATCAACAAAATACTCCTATTGGAGATGGACCAGTACT<br>TTTACCAGACAACCATTACTTATCAACACAATCTACCTTATCAAAAGATCCAAATGAAAAGAGAGA<br>TCACATGGTGTTATTAGAGTTTGTAACTGCTGCTGGAATTACACATGGCATGGATGAATTATACAA<br>AGCTAGCTCAGGAGGAGGGTCAGGAGGATCAGGGGGAGGATCCACTAGTCAAGGGAGCTCA<br>GCTGCAGGGAATTCTGGACAATTGTCCCATATGGCCCAACATGCTGGAGATAGTGCAGAAAATG<br>AAATTCAAGAGGAAAGAACTCAAGGTGAATAATTTGAAAATTGGAAGCAAAATGTGCCTTTTATG<br>TATGAAATCTGCATTAGCCATTAAAATTCATGGCCAAGCCTGACTGTAACTTGGTTGAATGAAATA<br>GAAATTGACTAAAATAATAACGAAGTACATAAATTGATTGTAGCTACTTAAACTGCCAGATAAGAA<br>CAAGAATTCATAAATGTACTTAAATTGTCACTTCCTTAGTATACAGAAGAAGATTTTGATGCTTCAA<br>GTTTAAATAACATATGGAAGACTTAACCTGTAGGTAAGATTACACAGGAATCACAAATACCAGTAT<br>AACATGAAATAAATAAGATAAGACAACAACCAATGTCTAAATCTATTCTGGCTGCTTAAACTTCTG<br>TTGGAGAAATATCCATATATGATATTAATAAACATCAAAAAGTAATGTCACTAAAAGGTTAAGAAAG<br>AGAGGGATATGGGTTGTCATGGAATCCTAAAAATTAAGGTCACCTATTGTCAGCAAGTTATGATA<br>AAAAGATTTATTACTGGGATGTAACAACTGGTTAATTGATAAAATCTTATAATTTTCATAGTTAAGAA<br>GTAGAAGATGTATGTTGGCATCCTCAAGATCCTAACCTATTTATATCATGTTCAGATGATAGAACT<br>TTTGCCATATGTGATACTAGGTCATAATAAGGAATGAAAATACAATAAGAAGCCCATTCTTAGGAA<br>ATTAATTGTATTTAATTCAATCAATTAGAACCAAGATATTTTGCTACAGGTTCTAATGATGCTGAAG<br>TAAAAATGTTCGACATCACTAAACCTGATAATTAAATTTACTCCTTTTCAAATCATGAAGATGCCAT<br>ATATACTCTTTAATGGTCTCCTCATAAAAAGAATTTGTTGGCTACAGGTTCTGTGGACAACAAAGT<br>TATCTTGTGGGATTATTTGAGAGTAGGTAAATCACAGGAGAGGGAATTTGAGAGAGATGGACCT<br>CCTGAAGTAGTGTTTTATCATGGGGGTCATAGATCGAAGGTGAATGATTTGTCTTGGAATCCTAA<br>TCATAAGAATTTGATGGCAAGTGTAGAAGCTGATAAAAATATGTTGTAGGTTTGGAAGATCTAACC<br>GTAATTGTGGATGGATGAAGATGGTGATGAAGCATGATTTATTATTAGTCTATATCTAACAATTTTA<br>TTCCAATTTTTATATTAATAATGTTCAAACTATCCAAATTGTTTTATTCATTCAAACATTCAAGTCGA<br>TCCAATATTTAACATAATCTGACATCCTTTAAAAAGAAAAGACGATAGGAATTACTCTATAACATTG<br>GTTTATACGGAGCTGCTTTTGGATGTGCCTTTGGTGCTGTCCCATTTTATAGATTGTGGTGTGAG<br>CATTTTGGCTTGGAGGGTGACCTTGATAAAAAGGACTATTCAATGAAAGGCAAAAAATGTATATC

TATCCTCTCATTTCTAGTGGATGTCTTCAGAAAATATCATATTGAATTTGGAGCAGAAACTGATCC
TGAAGCCAACTGGGAATTTC

The 5' flanking sequence of *Ku80c*, codon optimized GFP, recodonized *PtCAF1* and the 3' flanking sequence of *Ku80c* are in gray, green, yellow and purple respectively.

Table S4. Primers and *rRNA* probe sequences. Related to STAR methods.

	Name	Primers	
IES	Mating type IES	Forward	GGTGTTTATATCTTAATTGTTGACCCTCAC
		Reverse	CCATCTATACTCCATTCTTTATCTTAATTCAT
	51G11	Forward	ATCATAAGATTGATATCTTCTCCCTTCTCC
		Reverse	ACTTGCTACTAAAGCAAGAAACATTGAGAG
	51G1413	Forward	GAAGCTGCTTGTGTTAAGAATTCTACTGG
		Reverse	GCATCCAGCACTAGTTGAATTTACTGTAC
	51A712	Forward	TTTGTCAAAAAGACATGTATCAAAATGCAG
		Reverse	TAGAATACTAAGAGATTCAATACAACAAAC
	51A6649	Forward	ACTGCACCTCTAACTTTAACAAGCGAAGCA
		Reverse	CAGCAGTACATCCAGCTCTCTAAGTTTAGC
	51G2832	Forward	GCTATAACTCTTGAAGCTGCTTGTAATATG
		Reverse	TTGTCAATGAGCCATTAACAGTTGCTGGAT
	51G4404	Forward	CTGTTGCTACACATTGTGCATATGTTACT
		Reverse	GCTGTAAGATTAACATTGAGCATGATCAAG
	51A4578	Forward	CCTGCAGTAAGTTGCAGTTCAATAACTGG
		Reverse	TGTAGTCTTAAAATCTTAGCATGTTGTACC
	51A2591	Forward	ATGTGTTTGGACTGGATTGGCATGTAGAAG
		Reverse	GATGTAGCATAACATTTATCAACAATCCAT
	Dcl5d-01	Forward	CCAGTCTTTATAACTCCAAATATACTAATGTTAATTGCC
		Reverse	CTTGCTGGTTGAATATCAATTGAAAAATCTTGATG
	Dcl5d-02	Forward	TTTTCATACTCATCCTCACCCTACTCCC
		Reverse	ATAATATAAACTTTGAAGCCCCTGAAGCCG
Transposon	Thon	Forward	AGCGAACAGTTTATGGTAGTACCATTC
		Reverse	GACACTGAGTAGCTTGCCAATTTTCAA
	Sardine	Forward	GAACGCACTCGAAATGCAAGTGCTGC
		Reverse	GCCAGCTCATATAGGATAAAGAGTCTG
Actin		Forward	ATGTCTGAAGAACACCCAGCAGTCGTTATTG
		Reverse	ATCCCAATTATTGACAATACCATTATCAATTG
17s rRNA probe			ACC CGT GAC TGC CAT GGT AGT CCA ATA CA
